# The role of gamification in sustainable teacher education: Examining concept images of curriculum elements using a solomon four-group design

**DOI:** 10.1371/journal.pone.0346892

**Published:** 2026-04-30

**Authors:** Funda Uysal

**Affiliations:** Department of Curriculum and Instruction, Faculty of Education, Burdur Mehmet Akif Ersoy University, Burdur, Turkey; Farhangian Teacher Education University: Farhangian University, IRAN, ISLAMIC REPUBLIC OF

## Abstract

Despite the critical importance of curriculum literacy for effective teaching, developing pre-service teachers’ understanding of abstract curriculum elements remains a persistent and inadequately addressed challenge in teacher education worldwide. This research aimed to investigate the effects of gamification-supported teaching on pre-service teachers’ concept images (mental representations) of curriculum elements. The study employed a Solomon four-group experimental design (a robust design controlling for pretest effects) with 122 pre-service teachers studying at the Faculty of Education of a state university. Gamification activities based on individual and group work were implemented in the experimental groups (n = 61), while traditional teaching was used in the control groups (n = 61). Data were collected through a concept image questionnaire consisting of open-ended questions regarding curriculum elements (objectives, content, learning experiences, and evaluation) and student journals for reflective learning experiences from experimental group participants. The findings revealed that gamification-supported teaching had a substantive effect on concept images of curriculum elements, with large effect sizes for content (d = 1.59) and total score (d = 2.04). No significant pretest effects were observed, confirming methodological adequacy. Qualitative analysis revealed increased motivation (52 coded segments), enhanced conceptual understanding (47 segments), and interest-curiosity (43 segments) as primary themes. These findings demonstrate that gamification-supported flexible learning positively impacts both cognitive and affective outcomes. These results, consistent with similar studies in international literature, suggest that the gamification approach can be effective across different educational systems. These findings also have potential to contribute to the improvement of teacher education in achieving the “Quality Education” goal (SDG 4) within the United Nations’ Sustainable Development Goals framework. In conclusion, gamification-supported teaching can be utilized in teacher education as an effective approach for teaching abstract concepts such as curriculum elements. This research contributes to the teacher education literature by combining concept image theory with the gamification approach. Additionally, when evaluated from a sustainable teacher education perspective, the potential of the gamification approach as a pedagogical tool to support pre-service teachers’ long-term professional development has been demonstrated.

## Introduction

What is the role of education in achieving sustainable development goals? One answer to this question is that training qualified teachers forms the foundation for a sustainable future. So, how can we more effectively develop essential competencies such as curriculum literacy in teacher education? Traditional approaches sometimes remain limited in teaching abstract concepts like curriculum elements. At this point, gamification approach, which as Deterding et al. defined, is based on the principle of using game elements in non-game contexts, offers an alternative pedagogical framework [1]. As Kapp emphasized, gamification establishes a strong relationship between motivation and learning [2]. Various gamification elements such as badges, competitions, digital and physical activities can enrich the learning experience when used in individual and group work. In teaching abstract concepts, concept image theory -which examines how mental representations of concepts develop through formal and informal learning experiences- provides an important theoretical framework for understanding how gamification activities shape conceptual structures in students’ minds. This theory, introduced by Tall and Vinner, allows us to examine the development of pre-service teachers’ mental structures related to curriculum elements, contributing to our assessment of the potential of gamification approach in the context of sustainable teacher education [3].

Concept image is a complex phenomenon that includes all cognitive structures associated with a concept in an individual’s mind [3]. This cognitive structure encompasses not only the formal definition of the concept but also all mental images, properties, processes, and experiences related to that concept [4]. The formation of a concept image occurs as a result of the interaction of all formal and informal experiences related to the concept, and this process has a dynamic structure. As Sfard stated, the development process of concept image goes through the stages of internalization and condensation [5]. In this process, individuals first internalize the basic processes related to the concept, then transform these processes into more complex structures. The studies of Vinner and Dreyfus showed that the interaction between concept image and concept definition plays an important role in this development process [6]. Additionally, Kolb’s experiential learning theory emphasizes the importance of concrete experiences and reflective observations in the formation of concept image [7]. This theoretical framework provides a foundation for examining pre-service teachers’ concept images of curriculum elements and provides the theoretical basis for hypotheses (H₂, H₃) related to the pretest application.

Diversified learning experiences play an important role in the development of concept images. In this context, flexible learning environments support conceptual development by allowing students to progress at their own pace, adapt to different learning styles, and choose among various learning paths [8]. Gordon defined flexible learning as “an approach where learners have choices in terms of time, place, pace, and method.” [9]. When gamification is integrated with flexible learning principles, it provides a powerful pedagogical framework that increases learners’ motivation while adapting to their individual preferences [2,10]. The literature indicates that the integration of gamification and flexible learning offers potential for concretizing abstract concepts such as curriculum elements and responding to different learning preferences [11,12].

Research by Marton and Booth reveals the critical role of learning experiences in the development of concept image [13]. Especially in educational settings, learning experiences designed for the development of concept image should be organized in a way that enables individuals to understand and use the concept in different contexts. The importance of the development process of concept image in educational settings carries critical value especially in understanding and implementing the basic elements of educational programs. In this context, a deep understanding of the systematic structure of curriculum development process and curriculum elements is required.

Curriculum development is a systematic process built on Tyler’s four basic questions [14]. These questions are “What educational purposes should the school seek to attain?”, “How can learning experiences that are likely to be useful in attaining these objectives be selected?”, “How can learning experiences be organized for effective instruction?”, and “How can the effectiveness of learning experiences be evaluated?” These questions formed the basis for determining the basic elements of curriculum development: objectives, content, learning experiences, and evaluation. Taba expanded Tyler’s approach by proposing an inductive curriculum development model [15]. This model systematically addresses the determination of curriculum elements, starting from needs analysis and proceeding to identifying objectives, selecting and organizing content, organizing learning experiences, and evaluation processes. Taba’s approach is important in terms of emphasizing the dynamic relationships between curriculum elements. Ertürk, who made significant contributions to curriculum development studies in the Turkish education system, systematized curriculum elements as behavioral objectives, learning experiences, and testing situations in his curriculum development model [16]. This approach interpreted Tyler’s and Taba’s models in line with the needs of the Turkish education system, ensuring that curriculum elements are addressed in a coherent and dynamic structure. This theoretical framework on curriculum elements provides a basis for examining pre-service teachers’ concept images of curriculum elements and provides the conceptual foundation for the hypothesis (H₁) regarding the effectiveness of gamification.

The integration of flexible learning and gamification approaches offers significant opportunities in understanding the systematic structure of curriculum elements. Collis and Moonen defined five basic dimensions of flexible learning (time, content, entry requirements, instructional approaches, and resources) and emphasized that increasing learner control in each of these dimensions strengthens conceptual understanding [17]. Designing gamification-supported flexible learning environments in teaching curriculum elements supports the development of concept images of pre-service teachers by responding to different learning preferences [18,19].

Pre-service teachers’ competencies related to curriculum are gaining increasing importance in today’s global educational environment. The OECD’s “TALIS 2018 Results” report emphasizes that teachers’ curriculum knowledge and application is a fundamental competency area in 21st century education systems [20]. These competencies constitute an important dimension of pedagogical content knowledge as emphasized by Shulman [21]. As Bolstad stated, curriculum literacy is a fundamental competency area in teachers’ professional development [22].

The skills of understanding and implementing curriculum elements, as pointed out by Darling-Hammond, have a central position in the professional development of pre-service teachers [23]. According to UNESCO’s “Global Education Monitoring Report”, teachers’ abilities to understand and adapt curricula in different cultural and educational contexts are critically important for effective teaching [24]. The curriculum adaptation competency emphasized by Deng demonstrates the importance of pre-service teachers’ abilities to arrange curriculum elements according to different contexts and needs [25]. Henderson and Gornik’s [26] studies on curriculum leadership, highlight the importance of a continuous and systematic approach in the development of pre-service teachers’ curriculum literacy.

Innovative approaches are needed in the development of pre-service teachers’ concept images related to curriculum elements and in strengthening their curriculum literacy. In this context, gamification stands out as an effective approach in teaching curriculum elements. The educational foundations of gamification are based on the principle of using game elements in non-game contexts, as defined by Deterding et al. [1]. While Kapp [2] emphasizes the strong relationship between gamification, motivation, and learning, Werbach and Hunter [27] highlight the importance of systematically applying gamification principles in education.

The role of gamification and gamified learning environments in concept learning and deepening conceptual understanding is increasingly emphasized in international literature [11,28]. Domínguez et al. focus on how interactive learning environments transform the learning experience [29]. Gamification applications in teaching curriculum elements require systematic learning design, as stated by Nah et al. [30]. Zichermann and Cunningham’s [31] recommendations on the integration of game elements and Hanus and Fox’s [32] studies on assessment and feedback systems shed light on how gamification can be effectively used in teaching curriculum elements.

Recent international studies examine the educational effects of gamification in different cultural contexts. Hew et al. investigated the effects of gamification on learning outcomes in the Asian context and revealed that cultural factors could shape the effectiveness of gamification approaches [33]. Similarly, the European Commission’s “Digital Education Action Plan 2021-2027” framework provides a comprehensive perspective on how gamification can be applied in teacher education programs in different European countries [34]. These studies support the cross-cultural applicability of gamification approaches in education.

When examining the literature, it is seen that developing pre-service teachers’ concept images related to curriculum elements is important at a global level. Understanding the systematic structure of curriculum elements and their effective use has a critical role in the professional development of pre-service teachers [21,25]. However, the abstract nature of curriculum elements and their complex network of relationships can make it difficult for pre-service teachers to develop concept images related to these concepts. At this point, gamification stands out as an approach that increases motivation and deepens conceptual understanding in teaching curriculum elements [2,28]. Considering the importance of experiential learning in concept image development [7] and the role of gamification in creating interactive learning environments [29], examining the effects of a gamification-supported approach in teaching curriculum elements is important.

Although research examining the use of gamification approaches in teacher education has reached similar findings in different countries and education systems, studies directly examining the relationship between concept image development related to curriculum elements and gamification are limited. Tsay et al., in their study examining the effect of technology-supported gamification on learning experience, emphasized the effectiveness of gamification approaches in different cultural contexts [35]. Buckley et al. researched students’ perceptions of gamified learning and revealed its positive effect on motivation [36]. This research aims to fill this gap and contribute to the applicability of the findings in different cultural contexts. The research findings have the potential to contribute to global efforts to strengthen the curriculum literacy area emphasized in the teacher competency frameworks of international organizations such as OECD and UNESCO. A review of the literature reveals that developing pre-service teachers’ concept images of curriculum elements is important at both national and global levels. Additionally, this conceptual development is directly related to today’s vision of sustainable education.

Developing pre-service teachers’ curriculum-related competencies is closely associated with the United Nations’ Sustainable Development Goals (SDGs), particularly SDG 4 - “Quality Education.” As stated in UNESCO’s “Education for Sustainable Development” framework, educators need to adopt and implement innovative teaching approaches for a sustainable future [37]. Developing concept images of curriculum elements provides a foundation for pre-service teachers’ sustainable and long-term professional development. The gamification approach contributes to the development of sustainable learning practices by increasing pre-service teachers’ motivation and encouraging their active participation [38]. In this context, innovative approaches that develop pre-service teachers’ concept images of curriculum elements are of great importance for sustainable teacher education. In light of this sustainable education perspective, researching innovative approaches that will develop pre-service teachers’ concept images of curriculum elements is among the priorities of today’s education systems.

Despite the growing interest in gamification in education, recent systematic reviews reveal significant gaps that limit our understanding and application of gamification in teacher education. Dicheva et al. [39] found that “insufficient evidence exists to support the long-term benefits of gamification in educational contexts” and “the practice of gamifying learning has outpaced researchers’ understanding of its mechanisms and methods”. Zeng’s [40] comprehensive meta-analysis of studies from 2008–2023, while showing promising results for student engagement, highlighted that methodological limitations persist across gamification research. More critically, Ramírez Ruiz et al. [41] systematic review of 90 interventions found that applied research on gamification focuses primarily on higher education, “leaving aside primary education” and highlighting “the lack of teacher training and continuous support from educational entities”.

Most significantly, no studies have been identified that integrate the well-established concept image theory [3] with gamification approaches specifically in teacher education contexts. While concept image theory has been successfully applied to build teachers’ mathematical knowledge, and gamification shows promise in educational settings, the intersection of these approaches for developing pre-service teachers’ curriculum literacy remains unexplored.

This study addresses these critical gaps by providing: (1) theoretical contributions – the first integration of concept image theory with gamification in teacher education, addressing the identified theoretical gap; (2) practical contributions – evidence-based gamification strategies for teaching abstract curriculum concepts, responding to the need for teacher training support; (3) research contributions – rigorous experimental evidence using Solomon four-group design to address methodological limitations in previous gamification research; and (4) administrative contributions – empirical foundation for policy decisions regarding innovative approaches in teacher education programs aligned with sustainable development goals.

**Fig 1:**
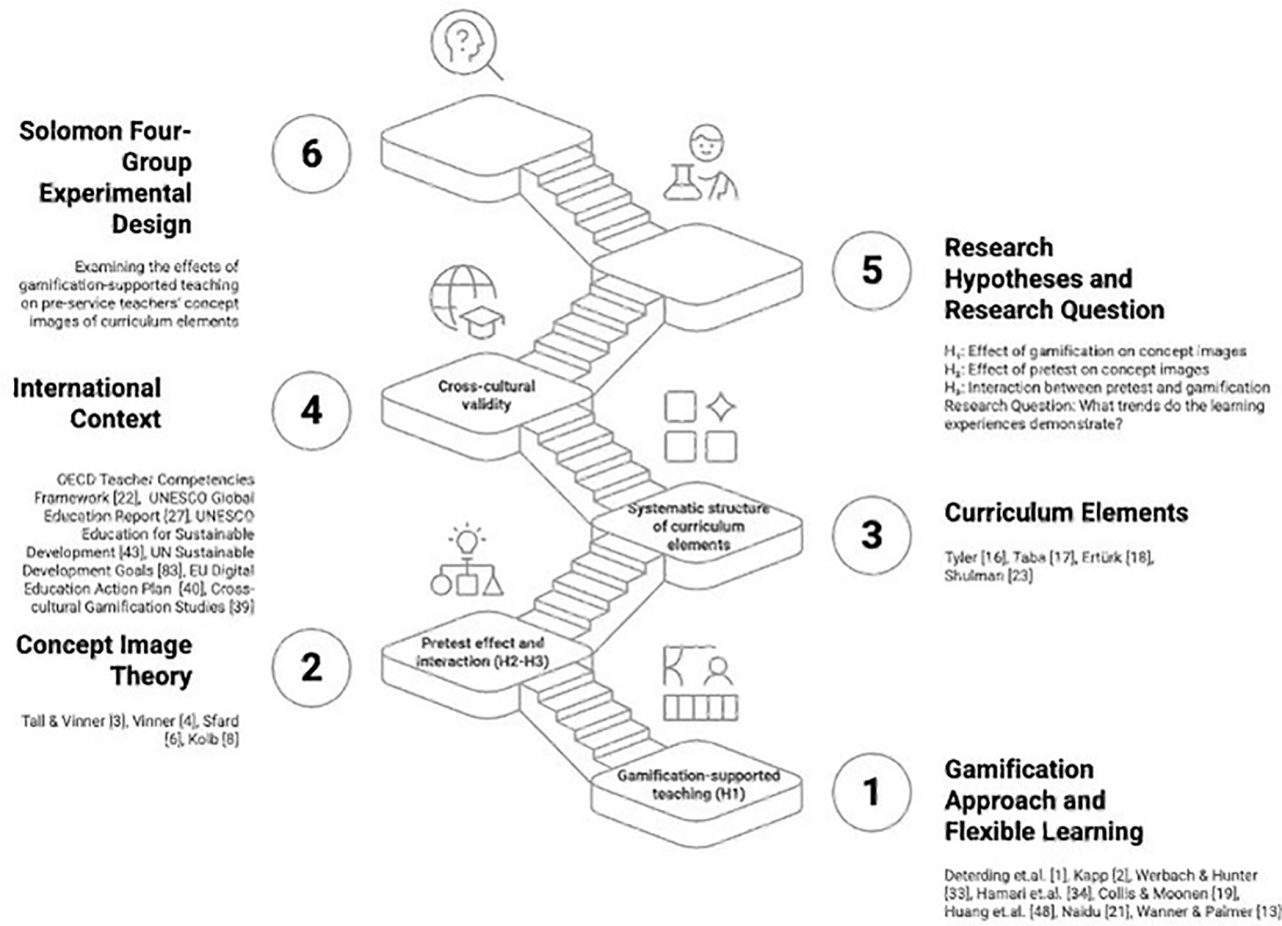
Conceptual framework of the research: Theoretical foundations, hypotheses, and research question.

In this context, the aim of the research is to examine the effects of gamification-supported teaching on pre-service teachers’ concept images of educational curriculum elements (objectives, content, learning experiences, and evaluation). In the research using a Solomon four-group experimental design, gamification-supported teaching was applied in the experimental groups, while non-gamification-supported teaching was applied in the control groups. In line with this theoretical framework and international research context, the conceptual framework presented below (Fig 1) illustrates the relationships between the research hypotheses and research question. The international context in the figure also includes UNESCO’s Education for Sustainable Development framework and SDG 4 (Quality Education) goals.

Based on the comprehensive literature review and theoretical framework presented above, the central research problem can be formulated as follows: While traditional approaches in teacher education often remain limited in effectively developing pre-service teachers’ understanding of abstract curriculum concepts, and although both concept image theory and gamification have shown promise in their respective domains, the integration of these approaches to enhance pre-service teachers’ concept images of curriculum elements remains unexplored. Specifically, it is unclear whether gamification-supported teaching can significantly improve pre-service teachers’ conceptual understanding of curriculum elements (objectives, content, learning experiences, and evaluation) compared to traditional non-gamification approaches.

Based on this research problem and grounded in concept image theory [3] and gamification literature [39–41], the following hypotheses were formulated to address different aspects of the research problem:

- H₁: Gamification-supported teaching has a significant effect on pre-service teachers’ concept images of curriculum elements.

This hypothesis directly addresses the core research problem regarding the effectiveness of gamification integration.

- H₂: Pretest application has a significant effect on pre-service teachers’ concept images of curriculum elements.

This hypothesis addresses potential sensitization effects identified in the methodology literature as important control factors.

- H₃: There is a significant interaction between pretest application and gamification-supported teaching.

This hypothesis examines whether the effectiveness of gamification varies depending on prior exposure to assessment, addressing methodological validity concerns.

To gain deeper insights into the research problem, the research also sought an answer to the question “What trend do the learning experiences reflected in the journals of pre-service teachers in the experimental groups show?” This qualitative component addresses the experiential dimension of the research problem by exploring how gamification-supported teaching influences pre-service teachers’ learning processes and perceptions, providing insights into the mechanisms through which gamification may enhance concept image development.

## Materials and methods

In this research, a Solomon four-group experimental design was used to examine the effect of gamification-supported teaching on pre-service teachers’ concept images of curriculum elements. The Solomon four-group design is a powerful experimental design as it allows for testing both pretest effect and the effect of experimental treatment simultaneously [42,43]. This design makes it possible to both control pretest sensitivity that threatens internal validity and test the effect of experimental treatment under different conditions [44].

The details of the Solomon four-group design are presented in Table 1.

**Table 1:** Solomon four-group design.

Group	Pretest	Treatment	Posttest
Experimental-1	O	X	O
Control-1	O	–	O
Experimental-2	–	X	O
Control-2	–	–	O

As seen in Table 1, the research included two experimental and two control groups, with a pretest applied to one experimental and one control group. Gamification-supported teaching was carried out in the experimental groups, while non-gamification-supported teaching was carried out in the control groups.

### Study group

The study group consisted of second-year students from the English Language Teaching and Primary Education programs at the Faculty of Education of a state university in the spring semester of the 2024–2025 academic year. Second-year pre-service teachers were specifically selected because this academic level represents the developmental stage when students are formally introduced to curriculum development concepts through required coursework. At this career stage, pre-service teachers are beginning to develop foundational understanding of curriculum elements (objectives, content, learning experiences, evaluation) but have not yet established deeply entrenched concept images, making this an optimal period for examining interventions designed to enhance conceptual understanding. The inclusion of two distinct teacher education programs (English Language Teaching and Primary Education) was intentional, providing initial evidence of gamification effectiveness across different subject-matter specializations within teacher education contexts. However, within these selected programs and academic level, existing classes were used in the research, and random assignment could not be made in the formation of the groups. Information about the study group is presented in Table 2. The study group consists of four groups in accordance with the Solomon four-group experimental design.

**Table 2:** Demographic characteristics of the study group.

	Experimental 1	Experimental 2	Control 1	Control 2	Total
Female	19 (63.3%)	22 (71.0%)	17 (54.8%)	22 (73.3%)	80 (65.6%)
Male	11 (36.7%)	11 (29.0%)	14 (45.2%)	8 (26.7%)	42 (34.4%)
Total	30 (24.6%)	31 (25.4%)	31 (25.4%)	30 (24.6%)	122 (100%)
Chi-Square	χ² = 2.849; p = .416				

As seen in Table 2, 65.6% of the study group consisted of female (n = 80) and 34.4% consisted of male (n = 42) pre-service teachers. There was no statistically significant difference between the four groups in terms of gender distribution (χ² = 2.849; p = .416). The fact that the experimental and control groups were equal in terms of both gender distribution and number of students is considered an important advantage in terms of healthy execution of the experimental procedure and ensuring internal validity [43]. Additionally, all teaching sessions across the four groups were conducted by the same instructor to eliminate potential instructor-related confounds, ensuring that observed differences could be attributed to the intervention rather than variations in teaching quality or style.

Participants were recruited through convenience sampling from existing classes in the spring semester of the 2024–2025 academic year. The convenience sampling approach was employed due to practical constraints inherent in educational research, where random sampling from broader populations is often unfeasible due to institutional requirements and classroom scheduling [43]. All second-year students enrolled in the specified programs were eligible to participate, with no additional exclusion criteria applied. While convenience sampling limits population generalizability, it was deemed appropriate for this experimental study as the primary focus was on testing causal relationships through controlled experimental manipulation rather than achieving population representativeness [43,44].

The Solomon four-group experimental design was implemented using existing class structures to balance methodological rigor with ecological validity [42]. The four groups were formed as follows: Experimental Group 1 (n = 30) and Control Group 1 (n = 31) consisted of English Language Teaching program students, while Experimental Group 2 (n = 31) and Control Group 2 (n = 30) comprised Primary Education program students. This assignment strategy maintained the integrity of natural classroom environments while fulfilling the requirements of the Solomon design for examining both intervention effects and potential pretest sensitization effects [42,45]. It should be noted, however, that this assignment resulted in a structural overlap between program membership and pretest condition, with both pretested groups (E1 and C1) drawn from the English Language Teaching program and both non-pretested groups (E2 and C2) from the Primary Education program. This overlap represents a methodological limitation that is addressed in the Research Limitations section.

The Solomon four-group design was specifically selected to address critical threats to internal validity, particularly testing effects that may occur when participants are exposed to pretest measures [42]. As Campbell and Stanley note, this design “provides both a replication of the experiment and a check on the external validity of the pretest” while controlling for “the interaction of testing and X” [42]. The design’s methodological strength lies in its capacity to separate true intervention effects from measurement artifacts, making it particularly valuable for educational interventions where pretest exposure could influence participants’ subsequent responses to treatment [45,46]. Although this design requires larger sample sizes and more complex implementation compared to simpler experimental designs, its ability to control for testing effects and provide multiple sources of evidence for treatment effectiveness justified its selection for this study [44].

Participant recruitment took place from March 17, 2025 to April 11, 2025. The study was approved by the Non-Interventional Clinical Research Ethics Committee of Burdur Mehmet Akif Ersoy University (decision no: GO 2024/796, approval date: December 3, 2024), and was conducted in accordance with the Declaration of Helsinki. All participants provided written informed consent prior to their inclusion in the study.

Additional ethical safeguards were implemented specifically for journal data collection. All participants provided specific informed consent for journal data use in research analysis, with explicit information that journal entries were voluntary research participation and not course requirements. Student journals were immediately anonymized upon collection using participant identification codes (E1-S01 format) and stored separately from consent forms and participant identification information. Participants were informed they could indicate any journal content they preferred not to be included in research analysis. The voluntary nature of journal reflection was emphasized throughout the implementation period, ensuring that non-participation would not affect academic standing or course evaluation.

### Data collection instruments and data collection process

In the research, a concept image questionnaire was used to determine pre-service teachers’ concept images of curriculum elements, and student journals were used to obtain the opinions of pre-service teachers about their experiences. The details of the instruments are as follows:

- *Concept Image Questionnaire:* The questionnaire consisted of open-ended questions regarding curriculum elements (objectives, content, learning experiences, and evaluation). Two questions were asked for each curriculum element. These were in the form of what the relevant curriculum element means and giving an example of the relevant curriculum element. For the draft form, a systematic expert review process was conducted. Three experts in curriculum development and one measurement-evaluation expert independently evaluated each item for relevance, clarity, and alignment with curriculum element dimensions. Expert consensus was achieved across all items, with strong agreement on item appropriateness and theoretical alignment. Additionally, one expert in Turkish language education was consulted for linguistic clarity. Based on expert feedback, minor revisions were made to question wording to enhance clarity while maintaining theoretical alignment with concept image framework.
  The draft questionnaire underwent pilot testing with 15 second-year pre-service teachers with characteristics similar to the intended study population. The pilot application served to confirm item comprehensibility, generate diverse response samples across conceptual understanding levels, and establish preliminary inter-rater reliability for scoring procedures. Pilot responses demonstrated appropriate variation in conceptual depth, ranging from superficial to comprehensive explanations, confirming the items’ ability to discriminate across understanding levels. Preliminary inter-rater reliability assessment using pilot data (two independent raters) demonstrated adequate scoring consistency, though rubric descriptors were refined based on scorer feedback to further enhance agreement. Based on pilot findings, the questionnaire was finalized without structural modifications, while minor clarifications were added to rubric performance level descriptors to optimize scorer reliability for the main study.
  The questionnaire’s construct validity was established through theoretical alignment with Tall and Vinner’s [3] concept image framework. The questionnaire operationalized this theory by assessing two fundamental dimensions for each curriculum element: (1) conceptual understanding (ability to define, explain properties, and articulate relationships), and (2) exemplification ability (capacity to generate contextually appropriate examples). This dual-dimension approach reflects the theoretical position that robust concept images require integration of definitional knowledge with practical exemplification capabilities [6]. The specific curriculum elements assessed represent universally recognized foundational components of systematic curriculum development [14–16], ensuring content domain representativeness.
  The decision to employ open-ended questions in the concept image questionnaire was grounded in established principles of qualitative research methodology and concept image theory. Open-ended questions were specifically chosen to capture the full richness and complexity of pre-service teachers’ concept images, which according to Tall and Vinner [3] encompass “all the cognitive structure in the individual’s mind that is associated with a given concept.” Closed-ended questions would have constrained participants’ responses to predetermined categories, potentially missing nuanced aspects of their conceptual understanding that are central to concept image development [47].
  Research in educational psychology demonstrates that open-ended questions allow participants to express their thoughts “in their own words, often leading to more in-depth and detailed responses” compared to structured alternatives [48]. This approach was particularly crucial for assessing concept images because, as noted in qualitative research literature, such questions “encourage exploration of a topic” and allow participants to “choose what to share and in how much detail” [49]. Furthermore, open-ended responses can reveal “unexpected insights” and “surprising mental models” that researchers might not anticipate when designing closed-ended alternatives [49]. Given that concept images are highly individual and can vary significantly among learners [3], the open-ended format was essential for capturing the authentic diversity of pre-service teachers’ conceptual understanding without imposing researcher-determined constraints on their responses.
  Given the open-ended response format requiring rubric-based scoring, traditional internal consistency coefficients were not applicable. For performance assessments scored with criterion-referenced rubrics, inter-rater reliability represents the most appropriate reliability evidence [50]. Inter-rater reliability was systematically assessed through independent scoring by two trained raters (both experienced curriculum development instructors) for all concept image protocols. As detailed in Table 5, multiple complementary reliability indices consistently exceeded acceptable thresholds across all curriculum elements and both pretest and posttest administrations (Fleiss Kappa: 0.60–0.88; Krippendorff’s Alpha: 0.60–0.88; ICC: 0.89–0.98), providing robust evidence of scoring consistency. It should be noted that one of the two raters was the researcher, who was therefore not blind to group membership (experimental vs. control) or measurement time (pretest vs. posttest). The second rater was an independent curriculum development instructor with no involvement in the study, and was consequently unaware of the group and time conditions under which responses were produced. While a formal blinding protocol was not implemented for either rater, the high inter-rater agreement achieved between a rater with full condition knowledge and a rater without such knowledge provides indirect evidence that scoring was guided by rubric criteria rather than by condition-related expectations. If expectancy bias had systematically influenced the researcher’s scoring, consistent agreement with an unaware independent rater at the levels observed (ICC: 0.89–0.98) would not be expected.
- *Student Journals:* Students in the experimental groups were asked to keep a journal during the implementation process. They were asked to reflect on their learning experiences after the relevant topic and share their opinions on their cognitive and affective acquisitions in this context. They were enabled to reflect on changes in their understanding of curriculum elements and their opinions on gamification activities.
  The student journal data collection followed a structured protocol designed to capture authentic learning experiences while minimizing interference with natural learning processes. Journal entries were completed immediately following each gamification activity session throughout the six-week implementation period. Specifically, participants in the experimental groups were asked to write reflective entries within 24 hours of each teaching session to ensure optimal recall and authentic reflection on their learning experiences [51]. This timing was strategically chosen based on research indicating that immediate post-experience reflection captures more detailed and accurate accounts of learning processes compared to delayed reflection [52].
  The journal prompts were intentionally kept broad and open-ended to encourage authentic self-reflection without leading participants toward specific responses. Participants were provided with guiding questions such as “Reflect on today’s learning activities and describe your thoughts, feelings, and observations about your understanding of curriculum elements” and “What changes, if any, did you notice in how you think about curriculum concepts after today’s activities?” This approach aligns with established practices in educational research where structured yet open-ended prompts support deep reflection while maintaining methodological rigor [53]. The asynchronous nature of journal writing allowed participants to reflect deeply and express their thoughts without time pressure, supporting the collection of rich qualitative data to complement the quantitative measures. Student journals were used as a qualitative data source to support quantitative data.

The research proceeded in line with the following learning outcomes: (1) Explains the basic elements of curriculum development (objectives, content, learning experiences, and evaluation) and the relationships between them. (2) Analyzes types of objectives according to cognitive, affective, and psychomotor domains. (3) Organizes program content according to different organization approaches. (4) Designs learning experiences considering learning principles and different teaching strategies. (5) Integrates measurement and evaluation approaches used in program evaluation.

**Fig 2:**
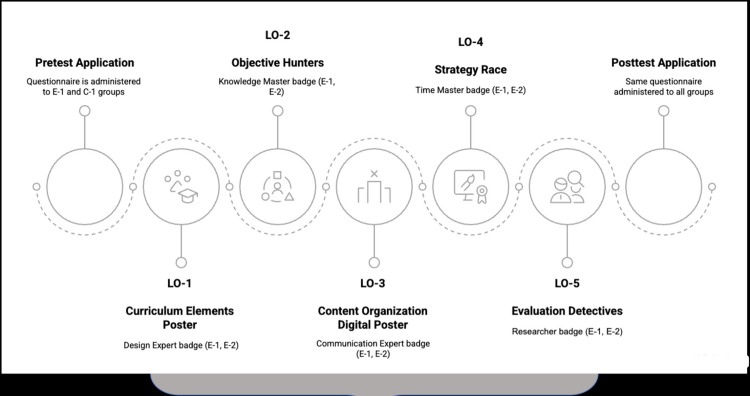
Data collection process.

The data collection process is shown in Fig 2.

The implementation carried out in the Curriculum Development in Education course lasted a total of seven to eight weeks, including pretest and posttest applications. As seen in Fig 2, in the first week, the concept image questionnaire was applied as a pretest to one experimental and one control group. Afterwards, teaching was carried out for six weeks. In the last week, the same concept image questionnaire was applied to all groups as a posttest. In the teaching process, the conceptual foundations of the relevant learning outcome were addressed using methods such as lecture, discussion, and question-answer in all groups. While teaching was supported by gamification in the experimental groups, it was not supported by gamification in the control groups. Gamification-based activities were designed and implemented for five basic learning outcomes in the experimental groups. In the “Curriculum Elements Poster” activity, designed to explain curriculum elements and the relationships between them, students working in groups of five prepared posters using colored cardboard, and the group that prepared the most creative and comprehensive poster had the chance to earn the Design Expert badge. In the “Objective Hunters” activity aimed at analyzing types of objectives, students competing individually on the Kahoot platform had a chance to earn the Knowledge Master badge based on their success, with the 5 highest scoring students being able to earn this badge. For implementing content organization approaches, students working in groups designed digital posters in the “Content Organization Digital Poster” activity. The group with the most effective communication was entitled to receive the Communication Expert badge. In the “Strategy Race” activity focusing on designing learning experiences, students competing individually through Google Form could earn the Time Master badge if they were among the 5 students who gave correct answers the fastest. Finally, in the “Evaluation Detectives” activity aimed at integrating evaluation approaches, the group that conducted the most comprehensive research had the opportunity to earn the Researcher badge.

**Fig 3:**
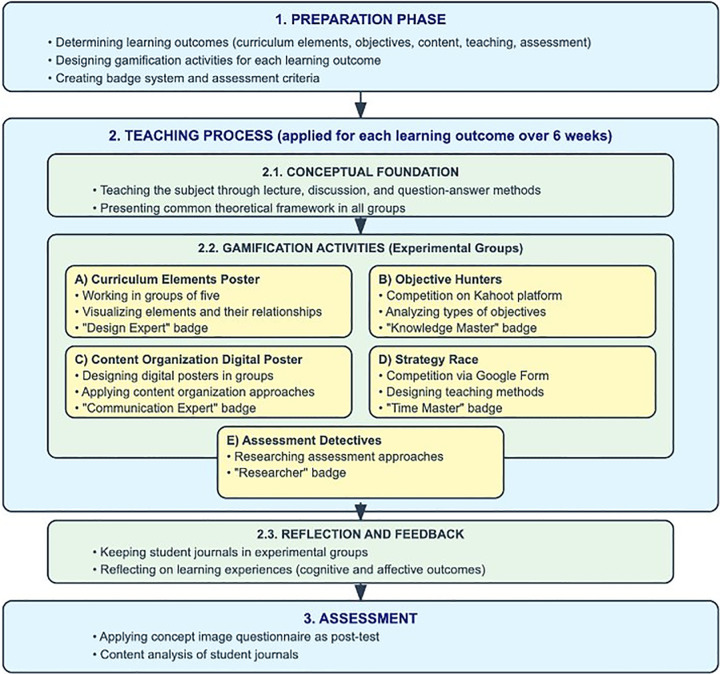
Gamification-supported teaching process.

The stages of the gamification-supported teaching process applied are visualized in Fig 3. This process, consisting of preparation, teaching, and evaluation stages, was designed to support the development of concept images of curriculum elements. The teaching process consists of establishing the conceptual foundation, implementing gamification activities, and reflective evaluation steps. Five different gamification activities were arranged to include both individual and group-based approaches for five basic learning outcomes. These activities were structured in accordance with the principles of flexible learning in practice.

After the data collection process, the stages of the gamification-supported teaching process applied are visualized in Fig 3. This process, consisting of preparation, teaching, and evaluation stages, was designed to support the development of concept images of curriculum elements. The teaching process consists of establishing the conceptual foundation, implementing gamification activities, and reflective evaluation steps. The five gamification activities applied include approaches based on both individual and group work in accordance with the principles of flexible learning. Curriculum Elements Poster, Content Organization Digital Poster, and Evaluation Detectives were based on group work, while Objective Hunters and Strategy Race were designed as individual activities. This arrangement supports the principle of suitability for learner preferences emphasized by Collis and Moonen in the flexible learning framework [17]. Digital platforms such as Kahoot and Google Form used in the activities allow students to progress at their own pace, strengthening the time and pace dimensions of flexible learning environments defined by Huang et al. [54]. The badge system implements the personalized feedback mechanism, which is one of Singh and Reed’s flexible learning principles, by defining different success criteria [55]. The reflection and feedback phase includes reflective practices emphasized by Naidu through student journals [19].

### Data analysis

In the data analysis process, a concept image assessment rubric for curriculum elements was first developed. The development of the rubric was based on two fundamental theoretical structures. The first is Tall and Vinner’s approach, which emphasizes that concept image is “all cognitive structure associated with a concept in an individual’s mind.” [3]. This approach was supported by Vinner’s [4] views that concept image includes definitional understanding and exemplification dimensions and Sfard’s [5] distinction between conceptual and operational understanding. The second theoretical foundation is the approaches of Tyler [14], Taba [15], and Ertürk [16], which reveal the systematic structure of curriculum development. In this context, the rubric aimed to evaluate the two main components of concept image—conceptual understanding and example-giving levels—by integrating them with the basic principles of curriculum development approaches. As suggested by Moskal and Leydens [46], the rubric criteria were written in a clear, understandable, and measurable way; indicators and sample expressions for each level were structured in line with the basic assumptions of curriculum development models. Following Reddy and Andrade’s [56] strategy for rubric validity, the opinions of three experts in the field of curriculum development were sought. Necessary adjustments were made to the rubric in line with expert opinions, and a pilot application was carried out. In accordance with Jonsson and Svingby’s [57] recommendations, the data obtained from the pilot application were analyzed and the rubric was finalized.

**Fig 4:**
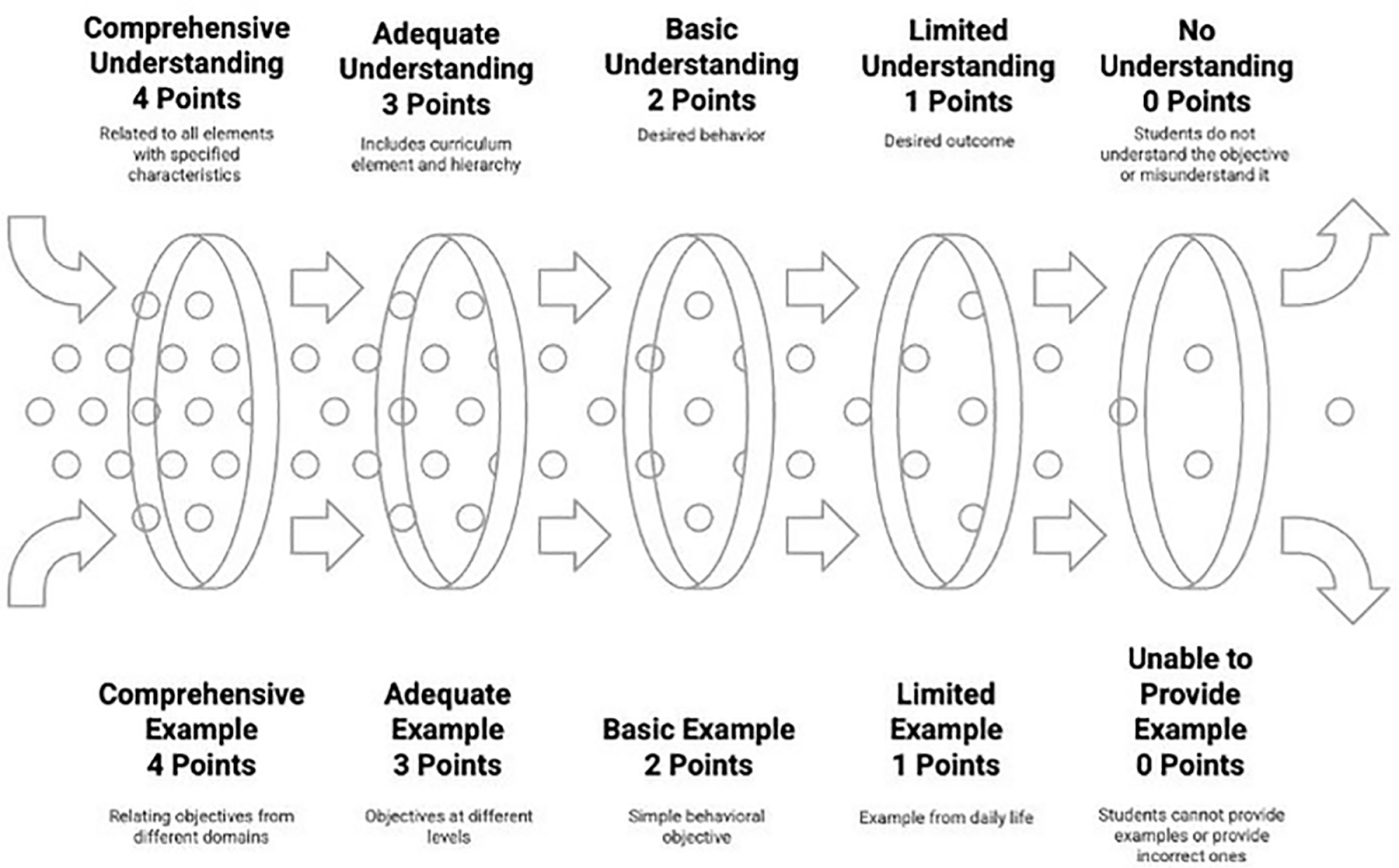
Levels for conceptual understanding and example-giving related to objective image.

Levels for conceptual understanding and example-giving related to objective image are shown in Fig 4.

As seen in Fig 4, the understanding and exemplification levels of the objective concept are evaluated separately from 0 to 4, creating five levels. At the 0-point level, students completely fail to understand the objective concept, using expressions like “I don’t know what objective means,” and give completely incorrect examples such as “student being good” in the example-giving dimension. At the 1-point level, there is a limited understanding; the objective is defined only as “desired outcome,” not seen as a curriculum element, and is limited to examples of objectives from daily life such as “being successful” in terms of example-giving. At the 2-point level, there is a basic understanding; the objective is defined as “desired behavior,” the measurability property is partially understood, and simple behavioral objectives such as “student performs addition operation” can be written. At the 3-point level, there is adequate understanding; the objective is positioned as a curriculum element, the distant-general-specific objective hierarchy is understood, and objectives at different levels can be written, establishing a hierarchy between objectives. At the highest level of 4 points, there is comprehensive understanding; the objective is related to other curriculum elements, systematically explained with all its properties, and objectives can be written in cognitive, affective, and psychomotor domains, and relationships between them can be established. This objective assessment rubric integrates multiple theoretical frameworks: Tyler’s [14] fundamental question “What educational purposes should the school seek to attain?” provides the systematic foundation for objective formulation criteria, while Taba’s [15] behavioral specification approach guides the example-giving dimension structure. The rubric incorporates concept image theory’s [3] emphasis on comprehensive cognitive structure assessment and Vinner’s [4] definitional understanding and exemplification dimensions. The 0–4 point progression reflects Sfard’s [5] conceptual understanding development stages from operational to structural comprehension, while criteria clarity and measurability follow Moskal and Leydens’ [46] rubric development principles.

**Fig 5:**
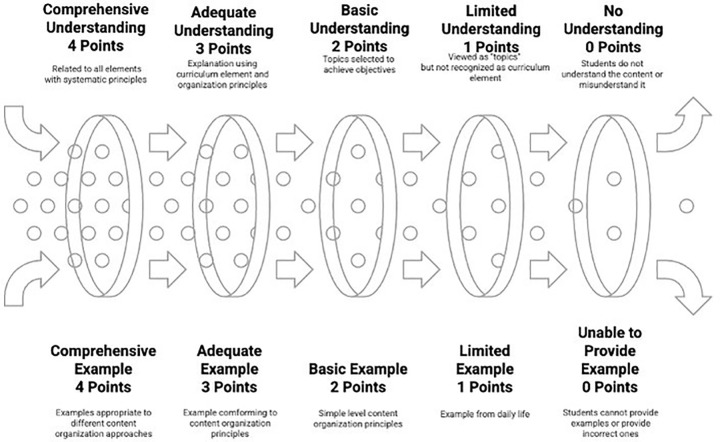
Levels for conceptual understanding and example-giving related to content image.

Levels for conceptual understanding and example-giving related to content image are shown in Fig 5.

As seen in Fig 5, the understanding and exemplification levels of the content concept are evaluated separately from 0 to 4, creating five levels. At the 0-point level, students completely fail to understand the content concept, using superficial expressions such as “content is what is covered in the lesson,” and give completely incorrect examples such as “what the student learns” or “textbook” in the example-giving dimension. At the 1-point level, there is a limited understanding; content is defined only as “topics,” not seen as a curriculum element, and is limited to examples of objectives from daily life such as “being successful” in terms of example-giving. At the 2-point level, there is a basic understanding; content is defined as “topics selected to achieve objectives,” basic organization principles are partially understood, and simple content organization examples such as “first natural numbers then fractions” can be given. At the 3-point level, there is adequate understanding; content is defined as a curriculum element, explained with organization principles, related to objectives, and examples according to content organization principles such as “from concrete to abstract” or “from simple to complex” can be given. At the highest level of 4 points, there is comprehensive understanding; content is related to other curriculum elements, organization principles are systematically explained, and examples according to different content organization approaches such as “spiral programming” or “linear programming” can be given, and interdisciplinary relationships can be established. The content rubric systematically integrates Tyler’s [14] content selection principles with Taba’s [15] inductive curriculum development model and Ertürk’s [16] Turkish curriculum framework for content organization. Concept image theory foundations [3] guide the assessment of content-related cognitive structures, while Vinner’s [4] definitional-exemplification approach shapes the dual evaluation dimensions. The hierarchical level system reflects Sfard’s [5] internalization and condensation processes, with rubric validation procedures following Reddy and Andrade’s [56] expert consultation strategy and Jonsson and Svingby’s [57] pilot application recommendations.

**Fig 6:**
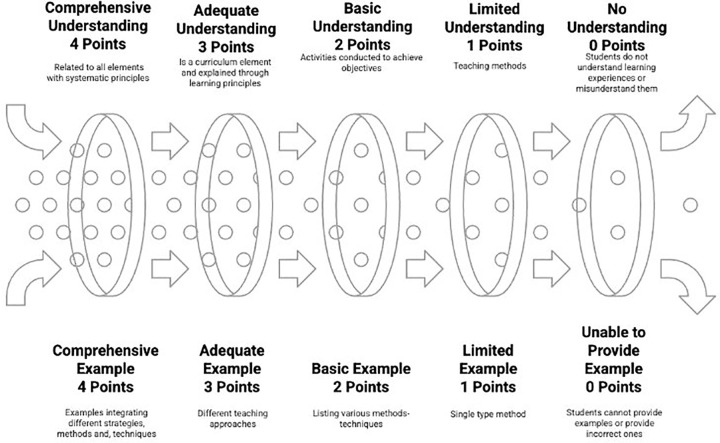
Levels for conceptual understanding and example-giving related to learning experiences image.

The levels for conceptual understanding and example-giving related to learning experiences image are shown in Fig 6.

As seen in Fig 6, the understanding and exemplification levels of the learning experiences concept are also evaluated separately from 0 to 4, creating five levels. At the 0-point level, students completely fail to understand the learning experiences concept, using superficial expressions such as “what is done in the classroom,” and give completely incorrect examples such as “students’ status” or “classroom environment” in the example-giving dimension. At the 1-point level, there is a limited understanding; learning experiences are defined only as “teaching methods,” not seen as a curriculum element, and is limited to single-type teaching method examples such as “we use direct instruction” in terms of example-giving. At the 2-point level, there is a basic understanding; learning experiences are defined as “activities done to achieve objectives,” basic teaching principles are partially understood, and various teaching methods-techniques such as “we use question-answer,” “we have students do group work” can be listed. At the 3-point level, there is adequate understanding; learning experiences are explained with learning principles, defined as a curriculum element, and activity examples according to different teaching approaches such as “preparing problem-based learning scenarios” can be given. At the highest level of 4 points, there is comprehensive understanding; learning experiences are explained systematically in relation to curriculum elements and with all teaching principles, and examples integrating different strategies, methods, and techniques can be given. These learning experience criteria embody Tyler’s [14] third fundamental question regarding learning experience organization effectiveness and Taba’s [15] systematic learning experience design principles. The rubric integrates Ertürk’s [16] behavioral objectives-learning experiences connection with concept image theory’s [3] experiential learning emphasis. Vinner’s [4] concept definition-image relationship guides the understanding-application assessment structure, while Sfard’s [5] process-object duality informs the progression from basic teaching recognition to comprehensive curriculum integration, implemented through Moskal and Leydens’ [46] clear measurement criteria.

**Fig 7:**
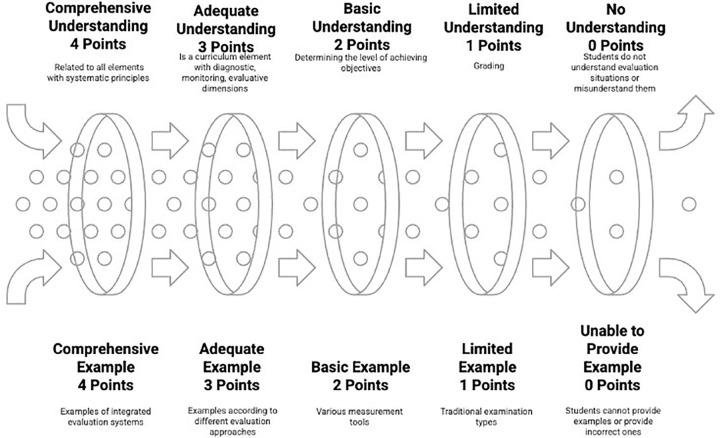
Levels for conceptual understanding and example-giving related to evaluation image.

The levels for conceptual understanding and example-giving related to evaluation image are shown in Fig 7.

As seen in Fig 7, the understanding and exemplification levels of the evaluation concept are also evaluated separately from 0 to 4, creating five levels. At the 0-point level, students completely fail to understand the evaluation concept, using superficial expressions such as “giving exams” or “assigning grades,” and give completely incorrect examples such as “student status” or “class success” in the example-giving dimension. At the 1-point level, there is a limited understanding; evaluation is defined only as “grading,” not seen as a curriculum element, and is limited to traditional exam types such as “we give written exams” in terms of example-giving. At the 2-point level, there is a basic understanding; evaluation is defined as “determining the level of achieving objectives,” basic measurement-evaluation principles are partially understood, and various measurement tools such as “preparing multiple-choice tests” can be suggested. At the 3-point level, there is adequate understanding; evaluation is defined as a curriculum element, the dimensions of diagnosis-monitoring-determining levels are explained, and examples according to different evaluation approaches such as “portfolio for process evaluation” can be given. At the highest level of 4 points, there is comprehensive understanding; evaluation is explained systematically in relation to curriculum elements and with all evaluation principles, and examples of integrated evaluation systems can be given. The evaluation rubric directly implements Tyler’s [14] fourth fundamental question about learning effectiveness assessment and Ertürk’s [16] testing situation specifications within the Turkish curriculum context. Taba’s [15] comprehensive evaluation approach guides the multi-dimensional assessment structure, while concept image theory [3] provides the foundation for evaluating evaluation-related cognitive structures. The rubric incorporates Vinner’s [4] definitional understanding assessment and Sfard’s [5] conceptual development stages, with validation procedures following Reddy and Andrade’s [56] expert review protocol and Jonsson and Svingby’s [57] empirical refinement recommendations.

In the rubric scoring process, as mentioned above, conceptual understanding and example-giving were evaluated separately for each curriculum element. The total score for each element consisted of the sum of conceptual understanding and example-giving scores. That is, it ranged from 0 to 8 points. In this context, a pre-service teacher who received full points for all elements received 32 points.

To test the research hypotheses, statistical analyses were performed on quantitative data, and content analysis was performed on qualitative data. Data analyses for the hypotheses are summarized in Table 3.

**Table 3:** Research hypotheses and analysis process.

Hypothesis	Dependent Variable	Independent Variable(s)	Participants	Analysis Method
H₁: Gamification-supported teaching has a significant effect on pre-service teachers’ concept images of curriculum elements.	Concept image scores for curriculum elements (objectives, content, learning experiences, evaluation, and total score)	Teaching approach (gamification-supported teaching, non-gamification-supported teaching)	All groups* (E1, E2, C1, C2)	Independent Groups t-test
H₂: Pretest application has a significant effect on pre-service teachers’ concept images of curriculum elements.	Concept image scores for curriculum elements	Pretest application (yes, no)	All groups (E1, E2, C1, C2)	2 × 2 Factorial ANOVA
H₃: There is a significant interaction between pretest application and gamification-supported teaching.	Concept image scores for curriculum elements	Pretest application × Teaching approach	All groups (E1, E2, C1, C2)	2 × 2 Factorial ANOVA
Research question: What trend do the learning experiences reflected in the journals of pre-service teachers in the experimental groups show?	Learning experiences	–	Experimental groups (E1, E2)	Content Analysis

*E1: Experimental Group 1 (English Language Teaching), E2: Experimental Group 2 (Primary Education), C1: Control Group 1 (English Language Teaching), C2: Control Group 2 (Primary Education).

Quantitative analyses were conducted using SPSS 28.0 [58], selected for its comprehensive capabilities in experimental design analysis, particularly Solomon four-group designs, and robust effect size calculations [59]. The software provided essential features for assumption testing, factorial ANOVA procedures, and Cohen’s d calculations with confidence intervals.

Qualitative content analysis was performed using Microsoft Excel 2019 with systematic coding procedures adapted from Elo and Kyngäs [53]. Excel was chosen for its transparency in coding procedures, accessibility for inter-rater reliability assessment, and proven effectiveness for thematic analysis in educational research contexts [60]. The Excel-based approach allowed for systematic organization of coding data with clear audit trails and collaborative coding procedures.

As seen in Table 3, to test the first hypothesis, independent groups t-test was applied over the posttest scores of all groups, comparing the experimental groups (E1, E2) with the control groups (C1, C2). In testing the second and third hypotheses, 2 × 2 factorial ANOVA analysis was used over the posttest scores of all groups. In this analysis, the factors of pretest application (yes/no) and teaching approach (gamification-supported teaching yes/no) were created, and the main effect of each factor and the interaction between factors were examined. The Solomon four-group design makes it possible to test both the effect of the experimental treatment and the possible effects of the pretest application and the interaction between these two simultaneously. To answer the qualitative research question, the journals of pre-service teachers in the experimental groups were examined using content analysis. This comprehensive analysis approach enhanced the validity and reliability of the research and allowed for the integration of quantitative and qualitative findings.

Before analyzing the data obtained in the research, normality and homogeneity of variances assumptions were examined to determine whether the use of parametric tests was appropriate.

#### Normality assumption.

To determine whether the data met the normality assumption, skewness and kurtosis values were examined. When examining the skewness and kurtosis values presented in Table 4, it is seen that skewness values range from −2.76 to 1.24, while kurtosis values range from −1.86 to 6.73.

**Table 4:** Skewness and kurtosis values for evaluating the normality assumption of the data.

Variable	Group	Skewness (Std. Error)	Kurtosis (Std. Error)
Objective	Experimental 1 (Pre)	0.06 (0.43)	0.27 (0.83)
Objective	Control 1 (Pre)	0.32 (0.42)	0.85 (0.82)
Content	Experimental 1 (Pre)	−0.55 (0.43)	−0.81 (0.83)
Content	Control 1 (Pre)	−0.13 (0.42)	−0.75 (0.82)
Learning Exp.	Experimental 1 (Pre)	0.43 (0.43)	−1.86 (0.83)
Learning Exp.	Control 1 (Pre)	0.48 (0.42)	−1.70 (0.82)
Evaluation	Experimental 1 (Pre)	−2.76 (0.43)	6.73 (0.83)
Evaluation	Control 1 (Pre)	−2.48 (0.42)	5.33 (0.82)
Elements-Total	Experimental 1 (Pre)	−0.75 (0.43)	0.32 (0.83)
Elements-Total	Control 1 (Pre)	0.01 (0.42)	−0.60 (0.82)

In general, skewness values within the ± 2 range and kurtosis values within the ± 7 range indicate that the data do not deviate excessively from normal distribution [61,62]. Deviation from normality was observed in only one variable (Evaluation pretest scores of the Experimental 1 group) (−2.76 skewness and 6.73 kurtosis). In this case, the use of non-parametric tests was considered, but taking into account that ANOVA is robust against deviations from the normal distribution assumption [63,64] and that parametric tests provide stronger results when group sizes are equal [65], it was decided to use parametric tests.

#### Homogeneity of variances.

The homogeneity of variances assumption was examined using Levene’s test. It was determined that the variances were not homogeneous for the Objective and Evaluation variables (p < .05), while the variances were homogeneous for the other variables (p > .05). In cases where variances were not homogeneous, Welch-corrected t-tests were used. This correction provides more reliable results in cases where variances are not equal [66,67]. In 2x2 ANOVA analyses, it is known that the F test is robust even when the homogeneity of variances assumption is not met when group sizes are equal [68].

### Validity and reliability of the research

The use of the Solomon four-group design ensured the control of factors that could threaten the internal validity of the research, such as maturation, test effect, and selection bias [42,45]. The standard implementation of the experimental treatment over a 6-week period (with the total research process spanning seven to eight weeks including pretest and posttest administrations) strengthened internal validity. Critically, all experimental and control group sessions were conducted by the same instructor to eliminate instructor-related confounds. This ensured that any observed differences between groups could be attributed to the gamification intervention rather than variations in teaching quality, pedagogical style, or content expertise. The instructor maintained consistent pedagogical approaches for conceptual foundations across all groups, with the only systematic difference being the presence or absence of gamification activities in experimental versus control groups, respectively. This instructor consistency, combined with the Solomon four-group design, contributes to internal validity controls [44]. Several structural features of the implementation reduced the risk of cross-group contamination. The four groups were enrolled in separate course sections and attended classes on different days of the week, limiting incidental contact between students across conditions. While these structural separations do not constitute a formal contamination control protocol, they represent meaningful practical barriers to the inadvertent sharing of instructional materials or activity content across experimental and control conditions. The inclusion of participants from different teacher education programs (English Language Teaching and Primary Education) increased the generalizability of the findings [69].

In the research, data triangulation was employed using concept image questionnaire and student journals. Expert opinion was sought and a pilot application was carried out in the development of the data collection instrument. The concept image assessment rubric for curriculum elements was developed based on Tall and Vinner’s [3] concept image approach and the curriculum development models of Tyler [14], Taba [15], and Ertürk [16].

Inter-rater reliability was examined as a result of the independent evaluations of two field experts in the rubric scoring. Fleiss Kappa, Krippendorff Alpha, and Intraclass Correlation Coefficient (ICC) were used to determine reliability. Table 5 presents the inter-rater reliability values.

**Table 5:** Inter-rater reliability values in rubric scoring.

	Pretest			Posttest		
	Fleiss Kappa	Krippendorff’s alpha	ICC	Fleiss Kappa	Krippendorff’s alpha	ICC
Objective	0.87	0.87	0.95	0.86	0.86	0.96
Content	0.88	0.88	0.95	0.83	0.83	0.97
Learning Exp.	0.83	0.83	0.95	0.84	0.84	0.97
Evaluation	0.74	0.74	0.89	0.79	0.79	0.95
Elements	0.70	0.70	0.96	0.60	0.60	0.98

As seen in Table 5, Fleiss Kappa and Krippendorff Alpha values range from 0.60 to 0.88. According to Fleiss’s [70] classification, values between 0.60–0.74 indicate good reliability, while values of 0.75 and above indicate very good evaluator reliability. Similarly, Krippendorff [71] stated that alpha values between 0.67–0.80 are satisfactory, while values of 0.80 and above are indicators of high reliability. ICC values range from 0.89 to 0.98. According to Koo and Li [72], ICC values between 0.75–0.90 indicate good reliability, while values of 0.90 and above indicate excellent reliability. These values show that the rubric scoring has high reliability.

Content analysis method was used in the analysis of student journals. The content analysis process was carried out in four stages: (1) coding the data, (2) creating themes, (3) organizing codes and themes, and (4) interpreting the findings [49]. In the coding process, two researchers first independently coded 10 journals and then came together to create a code list. All journals were coded in line with this list, and themes were determined. Two researchers independently coded 20% of journal entries (n = 37 from 183 total entries) to establish coding reliability. Inter-rater reliability was calculated using Miles and Huberman’s [73] formula [Reliability = Agreement/ (Agreement + Disagreement)]. The reliability coefficient obtained in the first independent coding was.82, disagreements on codes were discussed to reach consensus, and the final reliability coefficient was determined as.90, exceeding the 0.80 threshold recommended for educational research [47]. Additionally, the code and theme system was subjected to external auditing by an expert experienced in qualitative research, and necessary arrangements were made [74].

Qualitative data analysis was conducted using Microsoft Excel 2019, chosen for its systematic coding capabilities, transparency in inter-rater reliability procedures, and accessibility for collaborative coding [60]. Student journals were organized using structured Excel spreadsheets with participant identification codes (E1-S01 format), raw text segments, initial codes, theme classifications, and frequency tracking, ensuring transparent audit trails throughout the analysis process.

The detailed description of the experimental treatment and data collection process ensured the replicability of the research [75]. Furthermore, expert opinion was sought at every stage of the research process, and participant confirmation was made to increase the reliability of the findings [49]. For participant confirmation, the themes and interpretations formed as a result of content analysis were presented to 10 randomly selected students from the experimental groups, and final arrangements were made in line with their feedback. This process meets Tracy’s [76] criterion of “participant voice” for reliability and validity in qualitative research.

## Results

In this section, the results of statistical analyses performed for the research hypotheses and findings obtained from qualitative data are presented. Findings are presented and interpreted under separate headings for each hypothesis and research question.

### Results related to the first hypothesis: Effect of gamification-supported teaching

To examine the effect of gamification-supported teaching on pre-service teachers’ concept images of curriculum elements, the posttest scores of the experimental and control groups were compared using independent groups t-test. The results of these analyses are presented in Table 6.

**Table 6:** Experimental (posttest) – control (posttest) comparison.

Curriculum Element	Experimental	Control	t	p	Cohen’s d	95% CI
	Mean (SD)	Mean (SD)				
Objective*	4.9 (1.7)	3.2 (1.0)	6.74	<.05	1.22	[0.62, 1.82]
Content	5.0 (1.2)	3.2 (1.1)	8.78	<.05	1.59	[1.18, 2.00]
Learning Exp.	4.2 (1.5)	2.7 (1.1)	6.63	<.05	1.20	[0.61, 1.79]
Evaluation*	5.4 (1.2)	4.2 (0.9)	6.17	<.05	1.12	[0.55, 1.69]
Elements-Total	19.6 (3.2)	13.3 (2.9)	11.24	<.05	2.04	[1.60, 2.48]

* Welch t-test.

As seen in Table 6, statistically significant differences were found between the posttest scores of the experimental and control groups for all curriculum elements (p < .05). When examining effect sizes (Cohen’s d) [77], large effect size values (d > 0.80) were obtained for all curriculum elements, with 95% confidence intervals indicating large effects, though these estimates should be interpreted with caution as they do not account for clustering within intact classes (Objective: 95% CI [0.62, 1.82]; Content: 95% CI [1.18, 2.00]; Learning Experiences: 95% CI [0.61, 1.79]; Evaluation: 95% CI [0.55, 1.69]; Total: 95% CI [1.60, 2.48]). Particularly, the effect size obtained for the total score (d = 2.04, 95% CI [1.60, 2.48]) is large by conventional standards, suggesting a substantive effect of gamification-supported teaching that nonetheless warrants cautious interpretation given the individual-level analyses employed with intact class groupings. These findings show that gamification-supported teaching has a positive and practically meaningful effect on pre-service teachers’ concept images of curriculum elements. In line with this, the H₁ hypothesis was supported.

### Results related to the second and third hypotheses: Pretest effect and interaction

To examine the effect of pretest application and the possible interaction between pretest and gamification-supported teaching, a 2 × 2 factorial ANOVA analysis was performed. The results of these analyses are presented in Table 7.

**Table 7:** 2-way ANOVA to examine pretest-group interaction.

	Sum of Squares	df	Mean Square	F	p	Partial eta sq.
Objective						
Pretest	0.43	1	0.43	0.22	.64	0.002
Treatment	90.14	1	90.14	44.79	<.05	0.275
Pretest*Treatment	0.67	1	0.67	0.33	.57	0.003
Error	237.45	118	2.01			
Content						
Pretest	0.43	1	0.43	0.33	.57	0.003
Treatment	97.15	1	97.15	75.86	<.05	0.391
Pretest*Treatment	0.23	1	0.23	0.18	.68	0.001
Error	151.12	118	1.28			
Learning Exp.						
Pretest	0.59	1	.59	0.35	.56	0.003
Treatment	72.19	1	72.19	43.15	<.05	0.268
Pretest*Treatment	0.01	1	.01	0.01	.94	0.000
Error	197.40	118	1.67			
Evaluation						
Pretest	0.04	1	0.04	0.03	.86	0.000
Treatment	46.14	1	46.14	37.53	<.05	0.241
Pretest*Treatment	0.18	1	0.18	0.15	.70	0.001
Error	145.06	118	1.23			
Elements-Total						
Pretest	3.52	1	3.52	0.37	.55	0.003
Treatment	1199.91	1	1199.91	124.67	<.05	0.514
Pretest*Treatment	2.63	1	2.63	.27	.60	0.002
Error	1135.69	118	9.62			

As seen in Table 7, the pretest application did not have a statistically significant effect on any curriculum element (p > .05). Similarly, no significant interaction was observed between pretest application and gamification-supported teaching (p > .05). The fact that both effects show very low partial eta squared values (ηp^2^ < 0.01) indicates that the pretest effect and interaction effect are negligible in practice. On the other hand, the effect of the treatment (gamification-supported teaching) was confirmed to be significant for all curriculum elements (p < .05), and partial eta squared values (ηp^2^ = 0.241–0.514) indicate large effect sizes. Based on these findings, the H₂ and H₃ hypotheses were not supported.

These results provide important methodological advantages. The fact that the pretest effect is not significant and does not interact with the gamification application shows that the effect of the experimental treatment is not due to pretest sensitivity. This situation demonstrates that the effect of the experimental treatment (gamification-supported teaching) is consistent and strong regardless of whether a pretest was applied or not.

### Results related to the research question: Learning experiences reflected in the journals of pre-service teachers in the experimental groups

The data obtained from the journals of pre-service teachers in the experimental groups were analyzed using content analysis. As a result of the analysis, two main themes emerged: “Cognitive Acquisitions” and “Affective Acquisitions”. Findings related to these themes and their sub-themes are presented in Table 8.

**Table 8:** Content analysis results of student journals.

Main Theme	Sub-Theme	Frequency (f)	Expressions
Cognitive Acquisitions	Conceptual understanding	47	“I better understood the relationship between curriculum elements while preparing the poster.”
	Application ability	38	“Now I can consider taxonomic levels when writing objectives.”
	Analysis and synthesis	31	“I can compare content organization approaches.”
	Evaluation	26	“I can evaluate the strengths and weaknesses of different evaluation approaches.”
Affective Acquisitions	Increased motivation	52	“I studied more to earn badges.”
	Interest and curiosity	43	“I didn’t think I would find the curriculum development course this interesting.”
	Self-confidence development	35	“Now I believe I can explain curriculum elements.”
	Collaboration and interaction	39	“I learned a lot from my friends thanks to group work.”

The thematic analysis revealed a total of 305 coded segments across both main themes, with cognitive acquisitions accounting for 142 segments (46.6%) and affective acquisitions representing 163 segments (53.4%). This distribution indicates that gamification-supported teaching influenced both cognitive and affective domains substantially, with a slightly stronger emphasis on emotional and motivational dimensions. The detailed analysis of each theme and their constituent sub-themes is presented in the following sections.

#### Cognitive acquisitions theme: Hierarchical development pattern.

The cognitive acquisitions theme revealed a systematic hierarchical pattern of learning that aligns with educational psychology principles, demonstrating progressive cognitive development through gamification activities. This thematic structure reflects the scaffolded learning approach emphasized in concept image theory [3] and supports the cognitive development stages identified in Bloom’s revised taxonomy [78].

#### Primary level – conceptual understanding: Foundation building.

The most frequently reported cognitive acquisition focused on foundational understanding of curriculum elements, representing 33.1% of all cognitive-related journal entries. Pre-service teachers consistently emphasized improved grasp of abstract curriculum concepts through gamification activities. As one participant noted: “In the Curriculum Elements Poster activity, visually expressing the relationship between objectives, content, learning experiences, and evaluation situations helped me better understand the concepts” (E1-S12). This finding aligns with Tall and Vinner’s [3] assertion that concept image development requires rich cognitive associations formed through diverse learning experiences. The visual and interactive nature of gamification activities facilitated the formation of comprehensive mental structures that encompass both formal definitions and experiential understanding of curriculum elements.

#### Secondary level – application ability: Knowledge transfer.

Representing 26.8% of cognitive acquisitions, application ability emerged as the second most prominent sub-theme, indicating successful knowledge transfer from theoretical understanding to practical implementation. Pre-service teachers demonstrated enhanced ability to apply curriculum knowledge in realistic contexts: “In the Objective Hunters activity, I learned to distinguish different types of objectives. Now I can write objectives for cognitive, affective, and psychomotor domains” (E2-S7). This progression from conceptual understanding to application reflects Sfard’s [5] internalization process, where learners transform abstract concepts into operational knowledge. The gamification elements, particularly the competitive and badge-earning aspects, motivated students to practice and refine their application skills repeatedly.

#### Advanced level – analysis and synthesis: Critical thinking development.

Analysis and synthesis capabilities, comprising 21.8% of cognitive acquisitions, demonstrated the development of higher-order thinking skills through comparative and integrative activities. Pre-service teachers showed increased ability to examine curriculum elements critically and synthesize information from multiple sources: “In the Content Organization Digital Poster activity, I learned to analyze which approach would be more appropriate in which situations by comparing different content organization approaches” (E1-S23). This cognitive advancement supports Anderson and Krathwohl’s [78] revised taxonomy progression, where learners move beyond basic comprehension to analytical and creative thinking. The collaborative aspects of certain gamification activities fostered peer learning and multiple perspective integration, enhancing synthetic thinking capabilities.

#### Expert level – evaluation: Professional judgment formation.

The most sophisticated cognitive acquisition, representing 18.3% of cognitive themes, involved the development of evaluative judgment capabilities essential for professional teaching practice. Pre-service teachers demonstrated emerging expertise in making informed decisions about curriculum design and implementation: “When we researched and compared different evaluation approaches, my ability to decide which approaches would be more effective when creating an evaluation plan developed” (E2-S15). This highest level of cognitive development aligns with the expert-novice transition research in teacher education [79], where professional judgment emerges through guided practice and reflection. The research-based nature of activities like “Evaluation Detectives” provided authentic contexts for developing evaluative thinking skills crucial for future teaching practice.

This hierarchical cognitive development pattern demonstrates that gamification-supported teaching effectively scaffolds learning from basic conceptual understanding to expert-level professional judgment, supporting the theoretical framework’s emphasis on systematic concept image development.

### Affective acquisitions theme: Motivational and social development pattern

The affective acquisitions theme demonstrated a comprehensive pattern of emotional and social development that supports sustainable learning practices in teacher education. This thematic structure aligns with Deci and Ryan’s Self-Determination Theory [80], showing how gamification elements address basic psychological needs for autonomy, competence, and relatedness. The affective dimension proved equally important as cognitive development, representing crucial elements for long-term professional success in teaching.

#### Dominant pattern – increased Motivation: Intrinsic drive development.

Increased motivation emerged as the most prominent affective acquisition, representing 31.9% of all affective-related journal entries and demonstrating the central role of motivational enhancement in gamification effectiveness. Pre-service teachers consistently reported heightened engagement and sustained effort in learning curriculum concepts: “The thought of earning a badge encouraged me to study more. I studied types of objectives repeatedly especially to earn the Knowledge Master badge” (E1-S8). This finding supports Kapp’s [2] theoretical framework that gamification establishes a strong relationship between motivation and learning. The progression from extrinsic motivators (badges, competition) to intrinsic interest reflects the motivational internalization process described in Self-Determination Theory [80]. The sustained nature of this motivation, evidenced throughout the six-week implementation period, suggests that gamification elements successfully fostered enduring learning drive essential for lifelong professional development.

#### Engagement pattern – interest and curiosity: Academic engagement enhancement.

Interest and curiosity development, comprising 26.4% of affective acquisitions, indicated successful transformation of traditionally abstract curriculum topics into engaging learning experiences. Pre-service teachers reported unexpected enjoyment and spontaneous exploration of curriculum concepts: “Thanks to gamification activities, my interest in curriculum development topics that I might normally find boring increased. I especially started doing research on my own to learn about content organization approaches” (E2-S21). This transformation aligns with Csikszentmihalyi’s flow theory [81], where optimal learning experiences emerge when challenge levels match skill development. The gamification activities successfully created conditions for flow states by providing clear goals (badge earning), immediate feedback (competition results), and balanced challenges. The emergence of autonomous learning behaviors (self-directed research) demonstrates the development of scholarly dispositions crucial for professional growth in teaching.

#### Professional pattern – self-confidence development: Teacher identity formation.

Self-confidence development, representing 21.5% of affective themes, revealed the emergence of professional teacher identity and competence beliefs essential for effective teaching practice. Pre-service teachers demonstrated growing confidence in their ability to understand and communicate curriculum concepts: “I used to hesitate to talk about curriculum elements. Now I can easily explain these concepts to my friends” (E1-S4). This confidence building supports Bandura’s self-efficacy theory [82], which emphasizes the role of mastery experiences in developing professional competence beliefs. The progression from hesitation to confident explanation indicates successful scaffolding of learning experiences that built both knowledge and confidence simultaneously. This dual development is particularly significant for teacher education, as research consistently shows that teacher self-efficacy directly impacts instructional effectiveness and student outcomes [83].

#### Social pattern – collaboration and interaction: Professional learning community development.

Collaboration and interaction, comprising 23.9% of affective acquisitions, demonstrated the development of professional learning community skills essential for sustainable teacher education. Pre-service teachers valued the social learning opportunities created through group-based gamification activities: “Working with my group friends in the Curriculum Elements Poster and Evaluation Detectives activities allowed me to see different perspectives and produce more creative ideas” (E2-S11). This collaborative dimension aligns with Wenger’s communities of practice theory [84], which emphasizes learning as a fundamentally social process. The gamification design successfully created legitimate peripheral participation opportunities where pre-service teachers could engage in authentic professional discussions about curriculum elements. This social learning foundation supports the development of collaborative professional practices essential for career-long professional growth and school-based curriculum development initiatives.

The convergent pattern of affective acquisitions demonstrates that gamification-supported teaching creates conditions for holistic professional development, addressing not only cognitive learning but also the motivational, emotional, and social dimensions essential for sustainable teacher education and long-term professional effectiveness.

### Synthesis of findings: Integration and alignment with research framework

This synthesis integrates quantitative and qualitative findings to provide comprehensive evidence addressing the research hypotheses and question, while demonstrating alignment with the theoretical framework and contributions to sustainable development goals. The convergent evidence validates both the effectiveness of gamification-supported teaching and the methodological robustness of the research design, while establishing clear connections to international education quality initiatives.

#### Quantitative-qualitative convergence for hypothesis testing.

The convergence of statistical evidence and thematic analysis provides comprehensive validation of research hypotheses, demonstrating both the magnitude of gamification effects and the underlying mechanisms through which these effects occur. This dual-evidence approach strengthens the validity of conclusions and addresses multiple dimensions of the research problem.

#### Hypothesis 1 (H₁) – Comprehensive validation through dual evidence.

The hypothesis that gamification-supported teaching has a significant effect on pre-service teachers’ concept images of curriculum elements received robust support from converging quantitative and qualitative evidence. Quantitative analysis demonstrated large effect sizes across all curriculum elements (objectives d = 1.22, content d = 1.59, learning experiences d = 1.20, evaluation d = 1.12, total score d = 2.04), while qualitative findings revealed the underlying mechanisms through which these effects occurred. The cognitive acquisitions theme (142 segments, 46.6% of total qualitative data) directly explains how gamification activities facilitated concept image development through hierarchical progression from conceptual understanding (f = 47) to professional evaluation skills (f = 26). This convergence validates Tall and Vinner’s [3] concept image theory within gamification contexts and confirms the effectiveness of the pedagogical approach proposed in the conceptual framework (Fig 1). The triangulation of effect size evidence with thematic analysis provides robust validation rarely achieved in educational gamification research [85].

#### Hypotheses 2 and 3 (H₂, H₃) – Methodological robustness confirmation.

The non-significance of pretest effects (H₂: p > .05 for all curriculum elements) and pretest-treatment interactions (H₃: p > .05 for all curriculum elements) provides supporting evidence for methodological adequacy, validated by consistent positive learning experiences across all experimental participants regardless of pretest exposure. As Campbell and Stanley [42] emphasized, this finding strengthens the internal validity of the Solomon four-group design by confirming that treatment effects were not confounded by testing sensitivity. The very low partial eta squared values (ηp^2^ < 0.01) for both pretest effects and interactions indicate negligible practical significance, while treatment effects showed large practical significance (ηp^2^ = 0.241–0.514). Qualitative data supported this conclusion through uniform positive learning experiences across pretested and non-pretested groups, suggesting that gamification effects are not primarily attributable to pretest sensitization.

#### Research question response and theoretical framework validation.

The research question “What trend do the learning experiences reflected in the journals of pre-service teachers in the experimental groups show?” revealed a clear positive developmental trend characterized by systematic dual-domain growth. Cognitive development demonstrated hierarchical progression from basic conceptual understanding (f = 47, 33.1% of cognitive themes) through application ability (f = 38, 26.8%) and analysis/synthesis (f = 31, 21.8%) to evaluative judgment (f = 26, 18.3%). Simultaneously, affective development showed comprehensive engagement enhancement through increased motivation (f = 52, 31.9% of affective themes), interest-curiosity (f = 43, 26.4%), self-confidence (f = 35, 21.5%), and collaboration (f = 39, 23.9%). This dual-domain trend indicates that gamification-supported teaching creates conditions for holistic professional development essential for sustainable teacher education.

The identified developmental trends directly validate the theoretical relationships proposed in the conceptual framework (Fig 1). The connection between gamification approaches and concept image development is confirmed through the hierarchical cognitive pattern that mirrors Tall and Vinner’s [3] theoretical predictions. The integration of flexible learning principles is evidenced through diverse engagement patterns accommodating different learning preferences while maintaining pedagogical coherence. The sustainable teacher education dimension is validated through the development of both competence (cognitive acquisitions) and motivation (affective acquisitions) essential for long-term professional effectiveness.

#### Theoretical foundation integration.

The qualitative findings provide direct empirical support for Tall and Vinner’s [3] concept image theory within teacher education contexts. The hierarchical pattern of cognitive acquisitions demonstrates how gamification activities facilitated the formation of rich mental associations essential for robust concept images of curriculum elements. The progression from conceptual understanding to evaluative judgment aligns with Sfard’s [5] internalization and condensation processes, where students first internalized basic curriculum concepts through engaging gamification activities, then condensed these into operational knowledge for professional application. This theoretical validation extends concept image theory’s applicability beyond its original mathematical education context to teacher education domains.

The convergent evidence confirms established theoretical frameworks linking gamification with enhanced learning outcomes [2,28]. The dominance of increased motivation (f = 52) in qualitative findings explains the quantitative effect sizes, demonstrating how motivational enhancement serves as the primary mechanism through which gamification influences cognitive development. The progression from extrinsic motivators (badges, competitions) to intrinsic interest development supports Deci and Ryan’s [80] Self-Determination Theory predictions about autonomous motivation development. The balance between collaborative activities (collaboration f = 39) and individual challenges (application ability f = 38) reflects systematic application of gamification design principles emphasized by Werbach and Hunter [27] in educational contexts.

The findings validate the successful integration of gamification with flexible learning principles proposed in the conceptual framework. The diversity of both cognitive and affective acquisitions demonstrates how gamification activities accommodated different learning preferences while maintaining pedagogical coherence. Technology integration through digital platforms (Kahoot, Google Forms) successfully addressed Collis and Moonen’s [17] flexible learning dimensions, allowing personalized pacing and multiple engagement modalities. The emergence of autonomous learning behaviors (independent research initiation) supports Gordon’s [9] definition of flexible learning as providing learners with choices in time, place, pace, and method.

#### Contributions to SDG 4 (Quality education): Multi-level impact evidence.

The integrated findings demonstrate direct and substantial contributions to SDG 4 targets through converging quantitative and qualitative evidence, supporting sustainable teacher education as a pathway to quality education goals [37]. This contribution operates across multiple levels, from individual teacher development to systemic educational improvement potential.

Quantitative evidence demonstrates comprehensive improvement in curriculum literacy (total effect size d = 2.04), directly supporting SDG 4.c’s goal to “substantially increase the supply of qualified teachers” [86]. The particularly strong effect on content knowledge (d = 1.59) addresses critical curriculum competency gaps identified in OECD’s TALIS reports [20] and UNESCO’s global education monitoring research [24]. Qualitative evidence reinforces this contribution through demonstrated self-confidence development (f = 35), where pre-service teachers progressed from professional hesitation (“I used to hesitate to talk about curriculum elements”) to confident curriculum explanation (“Now I can easily explain these concepts”), indicating enhanced teacher readiness essential for quality education delivery. This confidence building aligns with research demonstrating that teacher self-efficacy directly impacts instructional effectiveness [23].

The research addresses SDG 4.7’s mandate to “ensure all learners acquire knowledge and skills needed to promote sustainable development” through evidence of sustainable learning practice development [86]. The affective acquisitions theme demonstrates cultivation of collaboration skills (f = 39), intrinsic motivation (f = 52), and autonomous learning behaviors essential for sustainable education approaches. The transformation from traditional passive learning to active engagement (“I especially started doing research on my own”) reflects the development of lifelong learning dispositions emphasized in UNESCO’s Education for Sustainable Development framework [37]. The development of collaborative professional practices supports the formation of learning communities essential for sustainable education implementation.

The methodological robustness demonstrated through the Solomon four-group design and consistently large effect sizes (d = 1.12–2.04) suggests that gamification-supported teacher education can serve as a scalable innovation for achieving SDG 4 goals internationally. The integration of accessible digital technologies (Kahoot, Google Forms) with evidence-based pedagogical approaches provides a sustainable model adaptable to diverse educational contexts globally. The cross-cultural validity potential is supported by similar findings in international gamification research [33,35], suggesting applicability across different educational systems. This scalability potential is particularly significant given the global teacher shortage and quality challenges consistently identified in UNESCO’s Global Education Monitoring Reports [24].

This comprehensive synthesis demonstrates that gamification-supported teaching represents a promising evidence-based approach for advancing SDG 4 goals through enhanced teacher education quality, with demonstrated immediate learning outcomes and substantial implications for sustainable education practice development globally.

## Discussion

### Interpretation of research findings

This research examined the effects of gamification-supported teaching on pre-service teachers’ concept images of educational curriculum elements using a Solomon four-group experimental design. The findings are interpreted in light of existing theoretical frameworks and empirical research.

#### Gamification effectiveness in concept image development (H₁).

The hypothesis that gamification-supported teaching has a significant effect on pre-service teachers’ concept images of curriculum elements was supported. The findings align with established theoretical frameworks and demonstrate the effectiveness of integrating game elements with educational content for abstract concept learning.

The high effect sizes observed across all curriculum elements can be explained by the multiple learning paths provided by gamification-supported flexible learning. When the flexible learning dimensions defined by Collis and Moonen are combined with gamification elements, they strengthen conceptual understanding [17]. Individual activities (Objective Hunters, Strategy Race) and collaborative activities (Curriculum Elements Poster, Evaluation Detectives) allowed students to progress according to their own learning preferences. Activities containing visual-auditory-kinesthetic elements proved effective in strengthening conceptual understanding, having the potential to appeal to students with different learning preferences. This finding is consistent with Wanner and Palmer’s conclusion that personalized learning experiences strengthen conceptual understanding [12].

The differential effects observed among curriculum elements can be associated with the nature of each element and the characteristics of the gamification activities used. The strongest effect observed for the content element can be explained by the “Content Organization Digital Poster” activity’s ability to visualize content organization approaches. This activity required students to visually express different content organization approaches (linear, spiral, modular, etc.), which strengthened the relationship between concept image and concept definition emphasized by Vinner and Dreyfus [6]. The relatively lower effect observed for evaluation may be related to the more complex structure of evaluation concepts and the “Evaluation Detectives” activity being more research-based. This finding suggests that different gamification strategies should be used in creating the image of complex concepts.

These results align with the findings from international meta-analysis studies conducted by Sailer and Homner [87], where gamification showed moderate to large effect sizes particularly on cognitive learning outcomes. Similarly, Huang et al. [54] revealed the positive effects of gamification on learning outcomes. Zainuddin et al. [88], who examined gamification effectiveness in teaching abstract concepts, demonstrated that game elements are effective especially in conceptual understanding and application-level learning. In international context, Hew et al. [33] in Asian education systems and Tsay et al. [35] in different higher education settings reached similar conclusions, showing that gamification can produce positive results across different cultural contexts.

However, it is important to consider that the large effect sizes observed may partially reflect Hawthorne effects (participants’ awareness of being in a study) or novelty effects inherent to gamified interventions. The introduction of gamification elements (badges, competitions, digital platforms) represented a pedagogical innovation for participants, potentially generating heightened engagement beyond the inherent educational value of these elements. As noted by Buckley et al. [36], initial enthusiasm for gamified approaches may diminish over time as novelty wears off. The six-week implementation period in this study may have captured peak novelty-driven engagement rather than sustained long-term effects. Longitudinal research tracking concept image development over multiple semesters would help distinguish genuine pedagogical effectiveness from transient novelty effects. Additionally, the very large effect sizes (d = 1.12–2.04) may partially reflect classroom-level clustering effects inherent to using intact classes as experimental units, as discussed in the Research Limitations section. Students within the same classroom may share unmeasured characteristics that amplify treatment effects beyond what might be observed in randomly assigned individuals. Cross-validation in more heterogeneous samples across multiple institutions would help determine whether these effects generalize beyond the current single-institution context.

#### Methodological robustness and validity (H₂, H₃).

The hypotheses regarding pretest effects and pretest-treatment treatment interactions were not supported, providing supporting evidence for methodological adequacy that strengthens the validity and generalizability of findings. These results show that the experimental treatment effects were not influenced by pretest sensitivity and that findings may be more broadly applicable.

As Campbell and Stanley stated, one of the strengths of the Solomon four-group design is that it allows for testing pretest effects and their interaction with the experimental treatment [42]. The non-significance of pretest effects and interactions in this research allowed for more reliable evaluation of gamification-supported teaching effectiveness. Shadish et al. emphasize that this kind of methodological robustness increases the replicability of similar research in different cultural contexts [44]. These results contribute to theoretical discussions on the effect of prior knowledge in learning processes and support the concept image theory connections established in the conceptual framework. It should be acknowledged, however, that the structural overlap between program membership and pretest condition introduces some ambiguity into these inferences, and the non-significance of H₂ and H₃ should therefore be interpreted with appropriate caution, as discussed in the Research Limitations section.

#### Learning experience patterns and professional development (RQ).

The research question examining learning experience trends revealed systematic professional development patterns that support both cognitive and affective dimensions of teacher preparation. The developmental trends demonstrate that gamification-supported teaching creates conditions for holistic professional development essential for sustainable teacher education.

Each gamification activity had different effects in teaching different curriculum elements. The “Curriculum Elements Poster” activity allowed students to visualize relationships between curriculum elements, contributing to holistic concept image development in line with the importance of learning experiences emphasized by Marton and Booth [13]. The “Objective Hunters” activity focused on objective writing skills development and enhanced the ability to write objectives in cognitive, affective, and psychomotor domains. This supported Sfard’s stages of internalization and condensation in the conceptual understanding process [5]. These activities proved effective in developing the “ability to integrate curriculum elements” emphasized in UNESCO’s global education report [24].

The qualitative findings support the positive effects of gamification-supported teaching on cognitive and affective acquisitions. The cognitive development themes show parallels with Bloom’s taxonomy levels [78] and reveal that gamification supports learning at different cognitive levels. As emphasized in Tall and Vinner’s concept image theory, concept image includes all cognitive structures associated with a concept in an individual’s mind [3]. The prominence of conceptual understanding in student journals demonstrates that gamification activities contribute to concept image formation.

The affective development themes are consistent with Kapp’s views on gamification’s effect on motivation [2]. The finding that gamification increases both intrinsic and extrinsic motivation, as shown in Koivisto and Hamari’s [89] study, supports these research results. The prominence of increased motivation in student journals shows that gamification elements (badges, competitions, points) are effective in enhancing motivation. This finding is consistent with van Roy and Zaman’s [90] study on gamification’s role in meeting basic psychological needs.

### Theoretical and practical implications

This section examines the broader theoretical contributions and practical applications of the research findings, connecting the empirical evidence to existing theoretical frameworks and identifying implications for educational practice and policy development.

The research findings offer significant theoretical contributions to concept image theory and gamification literature. Each gamification activity demonstrated distinct mechanisms for facilitating curriculum element understanding. The “Curriculum Elements Poster” activity enabled visualization of relationships between curriculum elements, contributing to holistic concept image development consistent with learning experience importance emphasized by Marton and Booth [13]. The “Objective Hunters” activity focused on objective writing skill development across cognitive, affective, and psychomotor domains, supporting Sfard’s internalization and condensation stages in conceptual understanding processes [5]. These activities proved effective in developing curriculum integration abilities emphasized in UNESCO’s global education frameworks [24].

The qualitative findings support positive effects of gamification-supported teaching on both cognitive and affective dimensions. The cognitive development themes show parallels with Bloom’s taxonomy levels [78] and reveal that gamification supports learning at different cognitive levels. As emphasized in Tall and Vinner’s concept image theory, concept image includes all cognitive structures associated with a concept in an individual’s mind [3]. The prominence of conceptual understanding in student reflections demonstrates that gamification activities contribute to concept image formation.

The affective development patterns are consistent with Kapp’s theoretical framework on gamification’s motivational effects [2]. The finding that gamification enhances both intrinsic and extrinsic motivation, as demonstrated in Koivisto and Hamari’s motivational information systems research [89], supports these results. The emphasis on increased motivation in student experiences shows that gamification elements (badges, competitions, points) effectively enhance engagement. This finding aligns with van Roy and Zaman’s research on gamification’s role in meeting basic psychological needs [90]. The most frequently emphasized themes in student reflections also demonstrate positive effects of flexible learning and gamification integration on cognitive and affective development. Singh and Reed’s “right person, right time, right way” approach in flexible learning design supports these findings [55]. Students’ ability to progress at their own pace on digital platforms (Kahoot, Google Form) aligns with Huang et al.’s principle of technology integration deepening conceptual understanding [54].

The gamification elements (badges, competitions, digital platforms, group work) and activities used in the research are consistent with Werbach and Hunter’s approach of dynamics, mechanics, and components in the gamification framework [27]. The transformation of extrinsic motivation elements such as earning badges into intrinsic motivation over time supports Deci and Ryan’s Self-Determination Theory [80]. The progression from external rewards to genuine interest in curriculum topics exemplifies this transformation. A similar transformation process has been observed in international gamification research by Buckley et al. [36] and Tsay et al. [35], indicating the cross-cultural similarity of motivational processes.

The conceptual foundations of gamification-supported teaching effects on concept images can be explained by Kolb’s experiential learning theory [7]. According to this theory, the learning process consisting of concrete experiences, reflective observations, abstract conceptualization, and active experimentation supports individuals’ concept image development. The gamification activities enabled students to have concrete experiences related to curriculum elements, reflect on these experiences, develop their conceptual understanding, and apply what they learned. This situation to developing systematic and holistic understanding emphasized in curriculum development approaches of Tyler [14], Taba [15], and Ertürk [16].

Regarding the use of gamification in teacher education, Gómez-Carrasco et al.’s [91] study showed the positive effect of gamification on pre-service teachers’ motivation and learning perceptions. The findings of this research also contribute to this literature by revealing that gamification is an effective approach in developing pre-service teachers’ concept images related to curriculum elements. The potential of gamification-supported teaching is particularly notable in developing the competencies emphasized by Shulman [21] in the context of pedagogical content knowledge and by Grossman [25] in the context of curriculum knowledge. This result is also consistent with the emphasis on innovative approaches in teacher education in OECD’s TALIS report [20].

The research findings offer applicable insights in different education systems. Gamification approaches that can serve the goals of strengthening digital competencies in teacher education emphasized in the European Commission’s “Digital Education Action Plan 2021-2027” framework were found to be effective in this research [34]. Additionally, in OECD’s TALIS 2018 report, the lack of innovative approaches in teacher education programs is identified as a significant problem in the comparative analysis of teacher education programs [20]. The results of this research offer a concrete approach to addressing this gap. These findings support the relationship between the international context component and the development of curriculum elements and concept images in the conceptual framework (Fig 1). Nevertheless, caution is warranted in generalizing these findings beyond the single-institution Turkish context. While the theoretical foundations (concept image theory, gamification principles) have demonstrated cross-cultural validity [33,87], and the curriculum elements examined represent universal educational components [24], the effectiveness observed may be influenced by contextual factors specific to this setting. The large effect sizes, while encouraging, require verification through multi-site replication studies employing random assignment to rule out potential confounds associated with institutional culture, student populations, or implementation-specific factors. Future research should examine gamification effectiveness across diverse cultural and institutional contexts to establish the boundary conditions and moderating factors that influence intervention success.

The research findings show that gamification-supported flexible learning environments are effective in teaching abstract concepts such as curriculum elements. Activities supported by digital technologies have allowed students to progress at their own learning pace and have experiences suitable for different learning styles. This flexibility is consistent with Bates’s principle of offering diversity in learning environments in the digital age [18]. The badge system has supported students’ progress according to their own strengths by defining different success criteria. In conclusion, the integration of gamification and flexible learning offers a supportive framework for the development of concept images in terms of both cognitive and affective aspects.

However, critical approaches to gamification use in education also exist. As Toda et al. [92] stated, gamification can also have negative effects. It is stated that extrinsic motivation elements may lose their effect over time, may increase competition among students, and may decrease the motivation of some students. Similarly, Buckley et al. emphasize that different student profiles may respond differently to gamification approaches [36]. The absence prominent negative effects in student journals in this research may be due to the balanced design of gamification. Nevertheless, it can be said that longitudinal studies are needed to evaluate the effects of gamification in the long term.

### Sustainable teacher education perspective

The findings of this research can be evaluated from a sustainable teacher education perspective in alignment with global education initiatives and long-term professional development goals. “Quality Education” (SDG 4), one of the Sustainable Development Goals, envisions providing inclusive and equal quality education for all students and promoting lifelong learning opportunities [86]. In this context, the gamification approach aimed at developing pre-service teachers’ curriculum literacy offers a sustainable pedagogical framework in terms of both motivational and cognitive acquisitions.

The substantive effectiveness observed in the research demonstrates the potential of gamification as a sustainable approach in teacher education. Particularly, the themes of “increased motivation” and “self-confidence development” that stand out in student journals align with the principle of “empowering students as active participants in their own learning processes” emphasized in UNESCO’s “Education for Sustainable Development Goals: Learning Objectives” report [37].

Gamification-supported teaching can contribute not only to developing pre-service teachers’ knowledge and skills but also to helping them acquire sustainable learning habits throughout their professional lives. As Evans et al. [93] state, sustainable teacher education requires approaches that support teachers’ continuous professional development and enable them to adapt to changing educational needs. The gamification-supported flexible learning environments implemented in this research have contributed to the development of lifelong learning skills by encouraging pre-service teachers to take initiative in their own learning processes.

Ferreira et al. emphasize that sustainable teacher education should be supported by innovative pedagogical approaches [94]. The gamification elements used in this research (badges, competitions, group work) created a more inclusive learning environment by addressing different learning preferences of pre-service teachers. This inclusivity aligns with the core principles of UNESCO’s vision for sustainable education. The theme of “Collaboration and interaction” mentioned in student journals can be considered as a dimension that contributes to the formation of sustainable learning communities.

As Redman states, sustainable teacher education should include not only environmental but also social and economic dimensions [95]. In this context, the social dimension of gamification-supported teaching was strengthened through collaborative learning environments, while its economic dimension was supported by the effective use of digital technologies. This integrated approach reflects the holistic nature of education for sustainable development.

The research results offer insights that can be applied in different cultural contexts. As Gómez-Carrasco et al. [91] emphasize, the effect of gamification in teacher education can yield similar positive results in different educational systems. This finding indicates that sustainable teacher education approaches can be shared and adapted globally.

In conclusion, the findings of this research reveal that gamification-supported teaching is a promising approach for sustainable teacher education, beyond strengthening pre-service teachers’ concept images of curriculum elements. The adoption of innovative and participatory approaches in teacher education in line with sustainable development goals will contribute to the global dissemination of quality education.

### Research limitations

**Fig 8:**
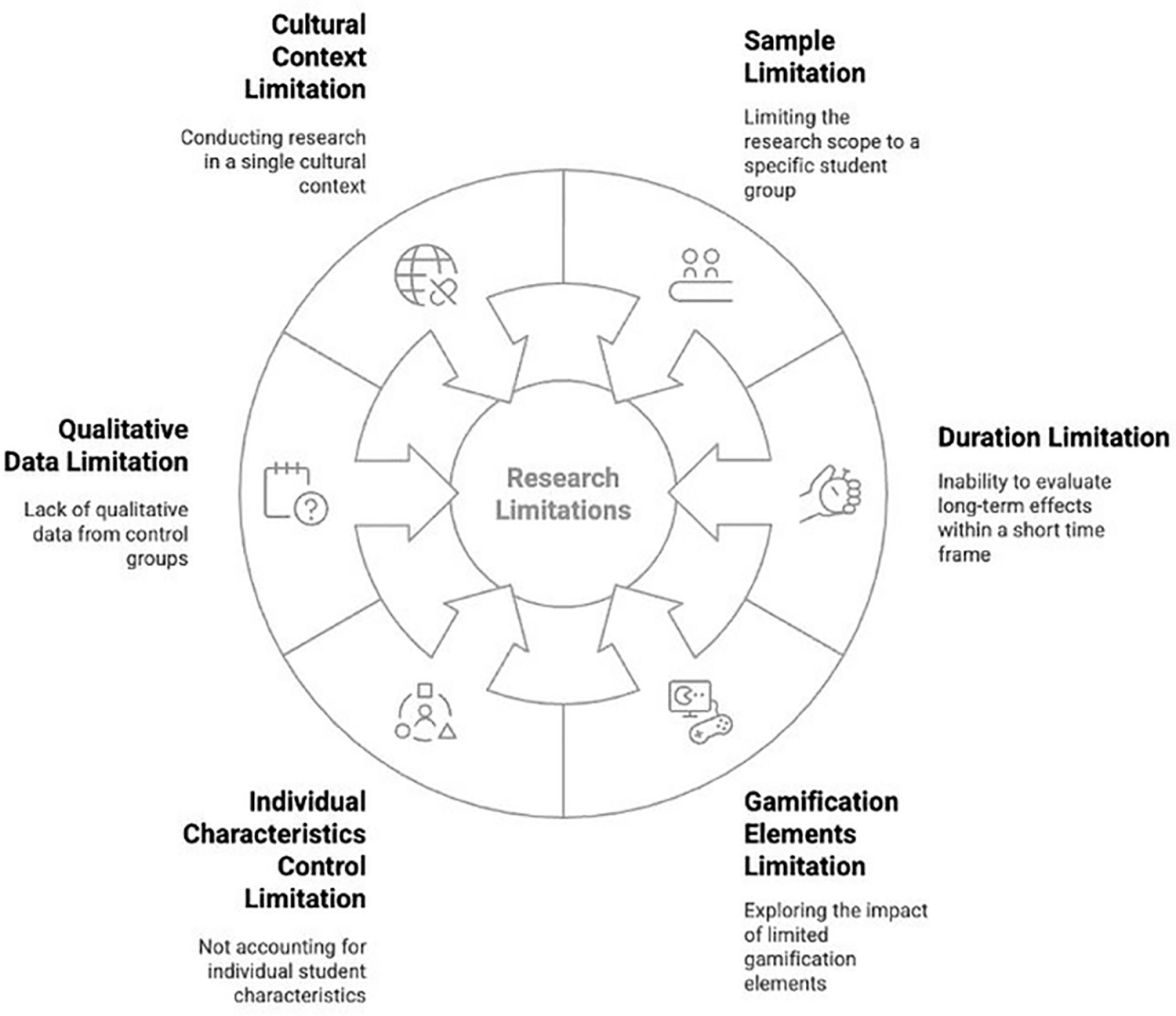
Research limitations.

Although this research shows the effectiveness of gamification-supported teaching, it has some limitations. These limitations are summarized in Fig 8.

As seen in the limitations in Fig 8, the research is limited to second-year students of English and Primary Education at a state university and covers a six-week implementation process. However, this timeframe aligns with standard educational intervention durations in experimental research and reflects practical constraints of academic semester structures [96]. The immediate effects observed provide a necessary foundation for potential long-term impacts, as concept image theory [3] suggests that robust mental structures formed through meaningful learning experiences tend to persist and influence subsequent learning. More importantly, substantial theoretical and empirical evidence supports the persistence and professional impact of the learning processes demonstrated in this study. The comprehensive nature of concept image development observed -spanning both cognitive acquisitions (conceptual understanding, application ability, analysis-synthesis, evaluation) and affective acquisitions (motivation, self-confidence, collaboration skills)- indicates deep structural changes in professional knowledge that are characteristic of durable learning. Research in teacher professional development demonstrates that intensive, theory-based interventions focusing on pedagogical content knowledge -such as our curriculum literacy development approach- produce lasting changes in teaching practice [23]. The self-confidence development documented in our qualitative findings represents the type of professional identity transformation that research indicates persists into teaching practice [83]. Furthermore, the autonomous learning behaviors observed in our study align with characteristics of self-directed lifelong learners essential for sustainable professional development [37]. The motivational transformation from extrinsic (badge-earning) to intrinsic interest in curriculum topics represents the type of motivational internalization that self-determination theory predicts will sustain engagement throughout professional careers [80].

An important methodological limitation concerns the restricted measurement scope employed in this study. While the research successfully measured concept image development through rubric-scored questionnaires and qualitative journal reflections—approaches well-aligned with concept image theory [3]—it did not incorporate complementary standardized measures that could provide multidimensional evidence of learning outcomes. Specifically, the concept image questionnaire assessed definitional understanding and exemplification abilities but did not measure cognitive processing depth through performance-based transfer tasks. We lack direct evidence of whether improved concept images enable pre-service teachers to apply curriculum knowledge in complex, authentic scenarios such as designing complete curriculum units, diagnosing curriculum design problems, or adapting curricula for diverse learners. Additionally, although student journals revealed themes of increased motivation, self-confidence, and interest, the study did not employ validated affective instruments such as the Intrinsic Motivation Inventory, Flow Experience Scale, or Teacher Self-Efficacy Scales that would enable quantitative assessment and comparison of motivational changes. The reliance on self-reported reflections, while providing rich qualitative insights, may be subject to social desirability bias or positive response bias. Furthermore, the study did not collect behavioral indicators such as digital trace data from gamification platforms (time-on-task, attempt patterns), task persistence metrics, or self-directed learning behaviors beyond required coursework. Without these behavioral measures, we cannot objectively verify whether self-reported increases in motivation translated into observable changes in learning behaviors. Future research should employ mixed-methods triangulation incorporating performance-based assessments in authentic contexts, validated pre-post affective measures, learning analytics from digital platforms, and delayed post-tests (3–6 months) to assess long-term retention and transfer. Such multidimensional assessment would strengthen confidence that observed concept image improvements reflect genuine, sustainable conceptual restructuring rather than transient measurement artifacts.

The use of intact classes without random assignment to experimental conditions may introduce selection bias despite the Solomon four-group design controls. The non-random assignment to experimental groups, necessitated by practical constraints of using existing classes, may confound results through classroom-level clustering effects. Students within the same classroom may share unmeasured characteristics (prior achievement levels, social dynamics, classroom culture) that amplify treatment effects beyond what might be observed with individually randomized participants. The very large effect sizes observed (d = 1.12–2.04), while statistically valid, may partially reflect such within-class similarities rather than purely intervention effects. Cross-validation in more heterogeneous samples with random assignment from larger populations would help verify whether these effects generalize beyond classroom-level characteristics and enhance confidence in causal attribution. Future research should employ random assignment where feasible and consider multilevel modeling approaches that account for clustering effects when intact classes are necessary. As a consequence, the standard errors, p-values, and confidence intervals reported in this study should be interpreted with appropriate caution, as individual-level analyses do not account for the non-independence of observations within intact classes and may therefore be overly optimistic.

A related but distinct methodological limitation concerns the structural overlap between program membership and pretest condition inherent in the group formation process. Because existing class structures were used, both pretested groups (E1 and C1) were drawn from the English Language Teaching program and both non-pretested groups (E2 and C2) from the Primary Education program. This means that program-level differences cannot be fully disentangled from pretest condition in the factorial ANOVA, which introduces ambiguity into the interpretation of H₂ and H₃ specifically, as the Solomon design’s pretest-related inferences are less definitive than would be achieved in a fully counterbalanced design where program membership was distributed across pretest conditions. The practical impact of this limitation is, however, partially mitigated by two considerations. First, neither pretest effects nor pretest-by-treatment interactions reached statistical significance for any curriculum element (p > .05), with negligible effect sizes (ηp^2^ < 0.01 throughout); if program-level differences had been operating as a confound masquerading as pretest effects, some degree of significance would be expected in these tests. Second, gamification effectiveness (H₁) was consistently observed across both programs, with large effect sizes in both the ELT groups (E1 vs. C1) and the Primary Education groups (E2 vs. C2), suggesting that the central finding is not program-dependent. Nevertheless, future implementations of the Solomon four-group design in educational research should aim to distribute program or departmental membership across pretest conditions to ensure cleaner separation of pretest effects from potential program-level influences [42,44].

A further consideration concerns the potential for cross-group contamination arising from the shared institutional context. Because all four groups were drawn from the same faculty and studied during the same semester, the possibility that students across conditions interacted informally and shared information about course activities cannot be entirely ruled out. It should be noted, however, that the four groups were enrolled in separate course sections and attended classes on different days, which provided structural barriers to such exchange. Furthermore, if contamination had occurred, its most likely direction would have been the transmission of gamification activity content from experimental group students to control group students, which would have artificially elevated control group posttest scores and thereby reduced rather than inflated the observed effect sizes. The consistently large effect sizes obtained in this study are therefore more consistent with the absence of substantial contamination than with its presence. Future research should implement explicit contamination prevention protocols, such as formally instructing participants to refrain from sharing course materials across sections, and where feasible, conducting experimental and control conditions in different academic terms to ensure more complete separation of conditions [44,45].

A further limitation concerns the absence of a formal blinding protocol in the rubric scoring process. As one of the two raters was the researcher, full blinding to group membership and measurement time was not achieved. Ideally, response protocols should have been presented to both raters in anonymized and randomized order, with group and time information concealed, to minimize the risk of expectancy bias. The high inter-rater agreement observed between the researcher and the independent rater (ICC: 0.89–0.98) provides indirect mitigation, suggesting that scoring was driven by rubric criteria rather than condition-related expectations. Nevertheless, future research employing rubric-based scoring should implement formal blinding protocols, presenting anonymized and randomized response protocols to all raters regardless of their involvement in the study.

The significant findings may partially reflect Hawthorne effects (participants’ awareness of being studied) or novelty effects associated with introducing gamification elements in a traditionally lecture-based curriculum course. Students’ heightened engagement and motivation may partially stem from the novelty of using digital platforms, earning badges, and participating in competitive activities rather than solely from the pedagogical value of these elements. As Hanus and Fox [32] demonstrated, positive effects of gamification can diminish over time as novelty fades. The introduction of gamification represented a pedagogical innovation for participants, potentially generating enthusiasm beyond the inherent educational value of game elements. The six-week implementation period may have captured peak novelty-driven engagement rather than sustained long-term effects. Longitudinal research tracking concept image development over multiple semesters would help distinguish genuine pedagogical effectiveness from transient novelty effects. Additionally, comparison groups exposed to equally novel non-gamified interventions would help isolate gamification-specific effects from general novelty effects. Future studies should include longer implementation periods spanning full academic terms or years and delayed post-tests to assess retention after novelty effects subside.

Since the gamification elements (badges, competitions, points) and activities used are limited to a specific framework, different gamification approaches may yield different results. However, this integrated approach was intentionally designed to reflect authentic gamification implementation contexts, where elements function synergistically rather than in isolation [97]. As Werbach and Hunter [27] emphasize in their comprehensive gamification framework, effective educational gamification requires systematic integration of dynamics, mechanics, and components rather than isolated element testing. Despite the integrated approach, our findings provide important insights into component effectiveness patterns. The differential effect sizes among curriculum elements reveal that content-focused gamification activities achieved the highest impact (d = 1.59), while the qualitative themes show that collaborative elements generated stronger ‘collaboration and interaction’ responses (f = 39) compared to individual competitive elements which primarily influenced ‘increased motivation’ (f = 52), suggesting complementary rather than competing functions. The badge system appeared to serve as an effective motivational scaffold, with student reflections indicating progression from extrinsic motivation (‘The thought of earning a badge encouraged me to study more’ - E1-S8) to intrinsic engagement (‘I especially started doing research on my own to learn about content organization approaches’ - E2-S21). This progression suggests that the badge component was successful in its intended function as a motivational catalyst rather than a long-term dependency. From a practical perspective, the integrated approach provides teacher educators with a complete, implementable framework rather than isolated techniques that would require additional integration work.

Additionally, individual characteristics of students (learning styles, gaming habits, digital literacy levels) were not considered as control variables. As Buckley et al. stated, individual differences can significantly shape the effectiveness of gamification [36]. In this context, the generalizability of the research findings is limited and needs to be replicated in contexts with different student profiles.

Another limitation is that qualitative data were collected only from the experimental groups. Collecting qualitative data from the control groups as well could have provided a more comprehensive understanding of the effect of gamification on learning experiences. As Johnson and Christensen emphasized, covering all groups in data triangulation in mixed method research increases methodological robustness [69].

Another limitation of the research is that it was conducted in only one cultural context with participants from a single state university in Turkey. However, multiple lines of evidence support the cross-cultural applicability of these findings. The theoretical foundation of this research enhances its generalizability potential beyond sample limitations. Concept image theory [3] and gamification principles have demonstrated cross-cultural validity in diverse educational settings, as shown by international meta-analyses [54,87]. The curriculum elements examined (objectives, content, learning experiences, evaluation) represent universal components of educational systems globally, as emphasized in UNESCO’s international curriculum frameworks [24]. The large effect sizes obtained in this research (d = 1.12–2.04) suggest robust treatment effects that typically transcend specific sample characteristics and indicate practical significance across contexts. Supporting our generalizability claims, similar gamification effectiveness has been documented across diverse cultural contexts including Asian educational systems [33], European teacher education programs [34], and North American higher education settings [35], suggesting that the fundamental psychological mechanisms underlying gamification effectiveness transcend specific cultural boundaries.

Despite these limitations, the strong experimental design of the research with the Solomon four-group design and the connections established with the theoretical framework have increased the reliability of the findings. As Shadish et al. stated, strong experimental designs support the replicability and generalizability of research findings [44]. In this context, the research results provide a solid foundation for conducting similar studies in different contexts.

## Conclusion and recommendations

This section presents conclusions and recommendations based on the research findings.

### Conclusion

**Fig 9:**
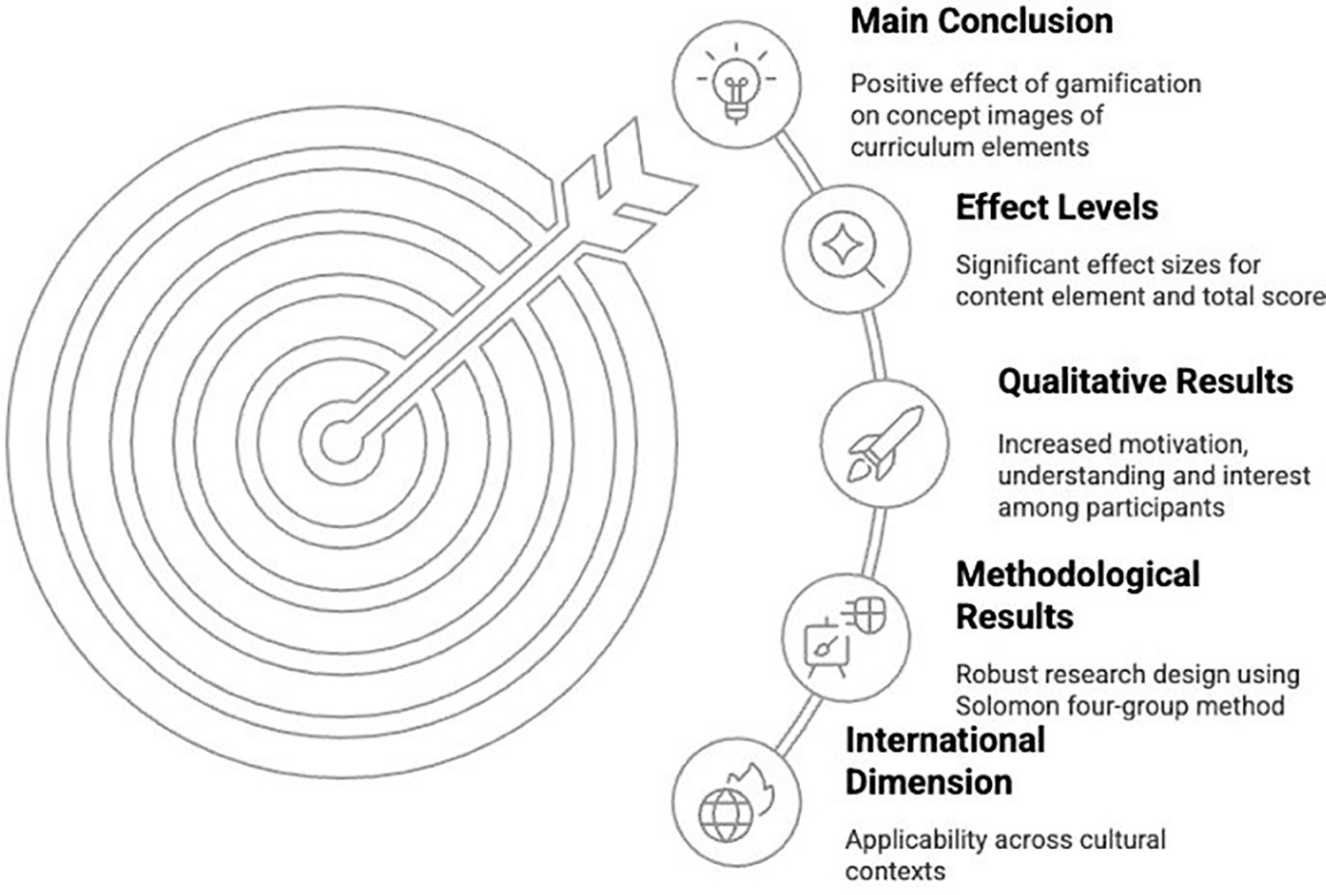
Research results.

This research examined the effects of gamification-supported teaching on concept images of curriculum elements and revealed the key findings shown in Fig 9. The research results have shown that gamification-supported teaching is an effective approach in developing pre-service teachers’ concept images of curriculum elements. When evaluated from a sustainable teacher education perspective, these findings demonstrate that gamification can be used as a sustainable pedagogical approach in teacher training programs. Considering the importance of teacher quality in ensuring access to quality education (SDG 4), strengthening pre-service teachers’ curriculum literacy will contribute to achieving sustainable development goals. The high effect sizes obtained in the research and the affective acquisitions highlighted in student journals demonstrate the potential of the gamification approach to support sustainable learning practices for pre-service teachers.

As seen in Fig 9, the research revealed that gamification-supported teaching has a positive and practically meaningful effect on pre-service teachers’ concept images of curriculum elements. Gamification was found to be an effective approach especially in terms of concretizing abstract concepts, increasing motivation, and cognitive-affective acquisitions. The largest effect sizes were obtained in the content element (d = 1.59) and total score (d = 2.04). In the qualitative findings, the themes of increased motivation (f = 52), conceptual understanding (f = 47), and interest-curiosity (f = 43) stood out.

One of the original aspects of the research is that it combines concept image theory with the gamification approach and aims to develop pre-service teachers’ understanding of curriculum elements in this context. The use of the Solomon four-group experimental design provided a methodological robustness rarely seen in gamification research. The research determined the differentiated effect sizes for each curriculum element and associated these differences with the characteristics of the gamification activities used.

The fact that the effects of gamification-supported teaching yield similar results in different cultural contexts and education systems suggests that this approach has the potential to serve the global education goals emphasized in the teacher competency frameworks of international organizations such as UNESCO and OECD. The main limitations of the research include the short-term nature of the research, being limited to certain gamification elements, and being conducted in a single cultural context.

### Gamification principles in teacher education

**Fig 10:**
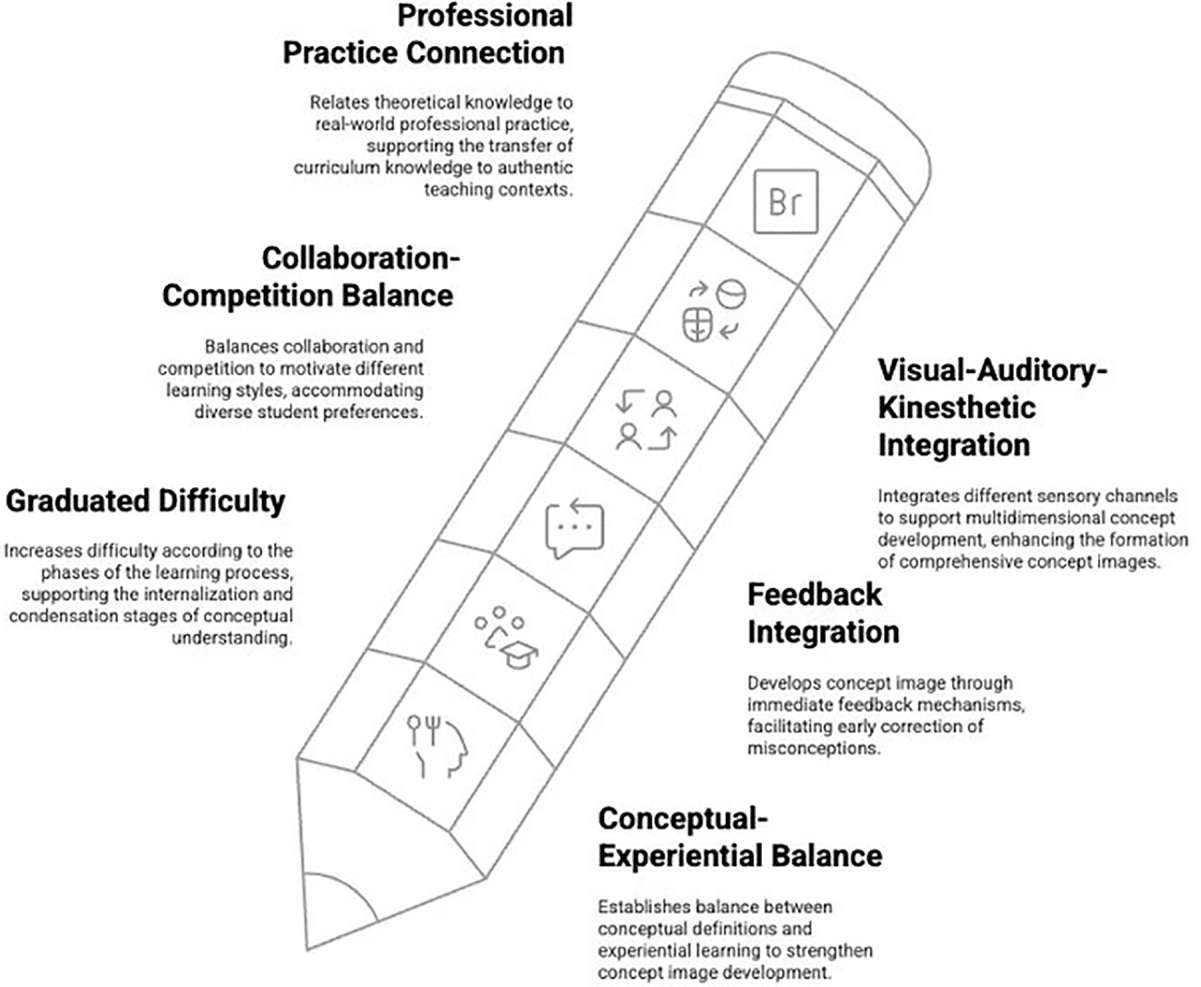
Gamification principles in teacher education.

Based on the research results, the gamification principles shown in Fig 10 can guide teacher educators on how to effectively implement gamification approaches in teaching curriculum elements.

As seen in Fig 10, gamification principles in teacher education are as follows:

- *Conceptual-Experiential Balance Principle:* A balance should be established between conceptual definitions and experiential learning activities in teaching curriculum elements. This balance strengthens the relationship between concept image and concept definition emphasized by Tall and Vinner [3]. The different effect sizes observed among curriculum elements (content: d = 1.59, objective: d = 1.22) and the expression in student journals “In the Curriculum Elements Poster activity, visually expressing the relationship between objectives, content, learning experiences, and evaluation helped me better understand the concepts” (E1-S12) show the importance of the balance between conceptual definitions and experiential activities.
- *Graduated Difficulty Principle:* Gamification activities should be designed from easy to difficult in accordance with the stages of the conceptual understanding process (internalization and condensation) of Sfard [5]. The expression in student journals “In the Objective Hunters activity, I learned to distinguish different types of objectives. Now I can write objectives for cognitive, affective, and psychomotor domains” (E2-S7) emphasizes the graduated progression under the theme of cognitive acquisitions (conceptual understanding [f = 47], application ability [f = 38], analysis and synthesis [f = 31]).
- *Feedback Integration Principle:* Immediate and continuous feedback mechanisms contribute to the development of concept image and ensure the correction of misunderstandings in early stages. The fact that “increased motivation” (f = 52) is the most frequently emphasized affective acquisition in student journals and expressions such as “the thought of earning a badge encouraged me to study more” (E1-S8) show the effect of immediate feedback on motivation.
- *Collaboration-Competition Balance Principle:* The balanced use of both collaborative and competitive gamification elements increases the motivation of students with different learning styles. Expressions such as “Working with my group friends in the Curriculum Elements Poster and Evaluation Detectives activities allowed me to see different perspectives and produce more creative ideas” (E2-S11) and “I studied hard to get the highest score in the Kahoot competition” emphasize the importance of both collaboration (f = 39) and competition elements.
- *Visual-Auditory-Kinesthetic Integration Principle:* Gamification activities targeting different sensory channels contribute to the multidimensional development of concept image. The fact that the highest effect size (d = 1.59) was observed in the content element and the expression “In the Content Organization Digital Poster activity, visually expressing different content organization approaches facilitated my understanding” show the effectiveness of activities targeting different sensory channels.
- *Professional Practice Connection Principle:* Gamification activities should be related to real practices of the teaching profession. This supports the principle of transferring theoretical knowledge to practice emphasized by UNESCO [24]. Expressions under the theme of self-confidence development (f = 35) such as “Now I can easily explain curriculum elements to my friends” (E1-S4) and “I used to hesitate to talk about curriculum elements” emphasize the importance of self-confidence development in terms of preparation for professional practice.

### Recommendations

**Fig 11:**
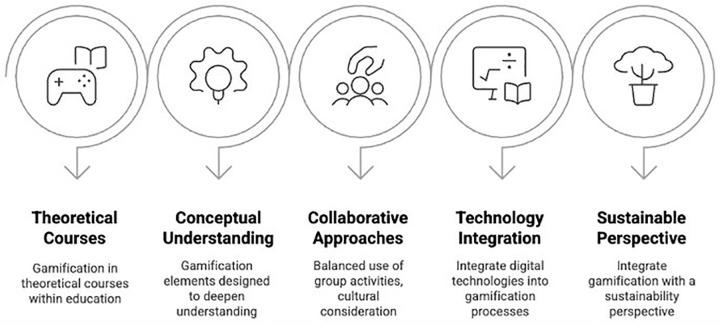
Recommendations for practitioners.

Recommendations for practitioners based on the research results are presented in Fig 11.

As seen in Fig 11, recommendations for practitioners are as follows:

- *Gamification in Theoretical Courses:* It is suggested that gamification-supported teaching practices be included in theoretical courses within teacher education programs. This recommendation is supported by UNESCO’s global education report emphasizing innovative teaching approaches [24].
- *Conceptual Understanding and Element-Focused Design:* Gamification elements can be designed to support cognitive processes aimed at deepening conceptual understanding and tailored to the nature of each curriculum element. The meta-analysis study by Sailer and Homner [87] and the systematic review by Dicheva et al. [11] emphasize the effectiveness of such approaches.
- *Collaborative and Culturally Sensitive Approaches:* A balanced use of individual and group-based activities in developing concept images, along with consideration of the cultural characteristics of the target audience, would be valuable. Differences in collaborative learning preferences among students from different cultural contexts can be taken into account [33,36].
- *Technology Integration and Reflective Practices:* The integration of digital technologies (Kahoot, Google Forms, mobile applications) into gamification processes and the inclusion of practices such as journal keeping that allow students to reflect on their learning experiences could be beneficial. The European Commission’s [34] digital education action plan and the studies by Tsay et al. [35] support these approaches.
- *Sustainable Teacher Education Perspective:* In line with SDG 4 (Quality Education) goals, consideration could be given to integrating gamification applications that include a sustainability perspective into teacher training programs and developing long-term learning strategies. Digital gamification platforms can be evaluated as sustainable and accessible learning resources in teacher education.

**Fig 12:**
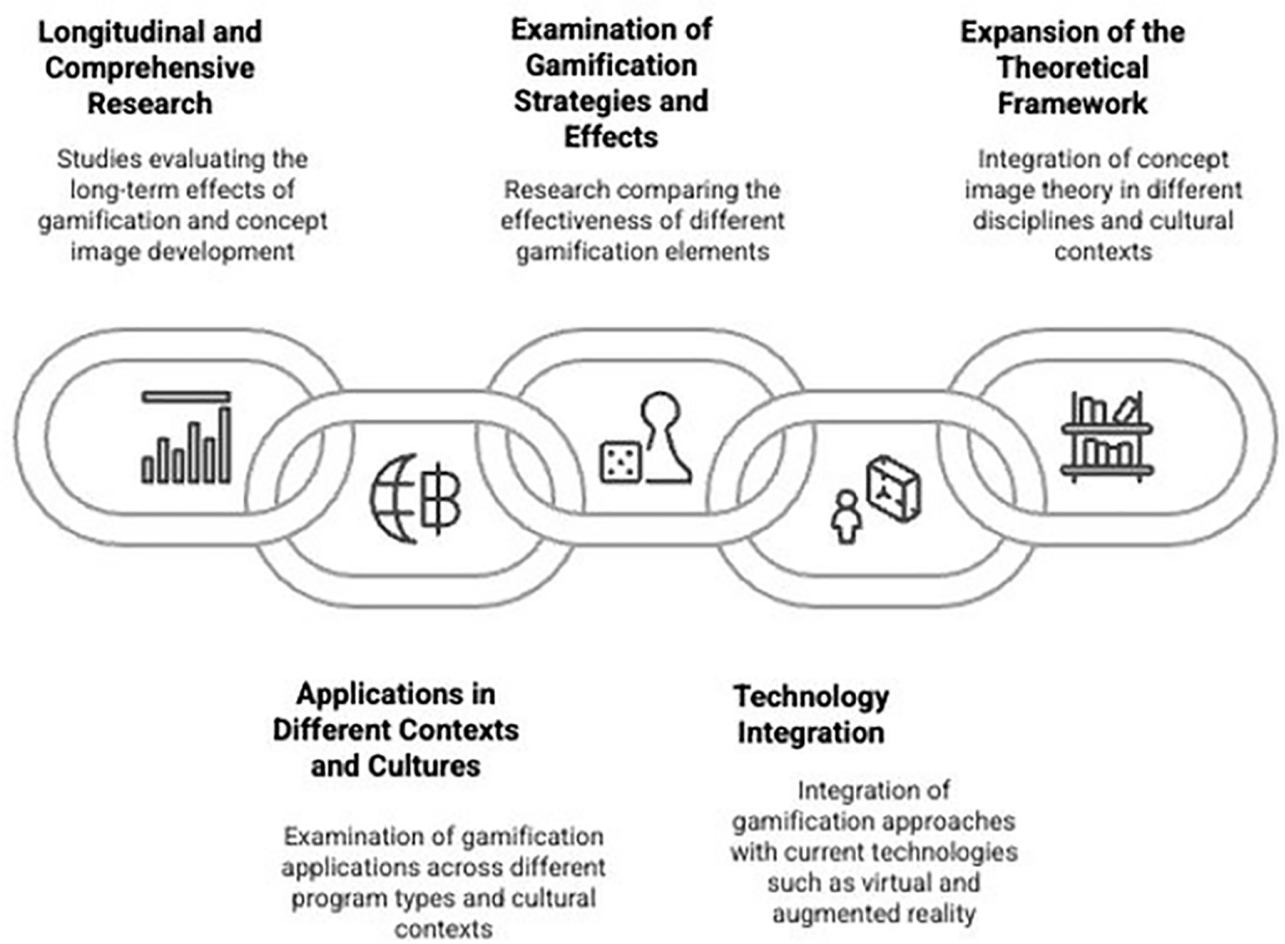
Recommendations for researchers.

Recommendations for researchers based on the research results are shown in Fig 12.

As seen in Fig 12, recommendations for researchers can be listed as follows:

- *Longitudinal and Comprehensive Research:*
  - Longitudinal Studies: Longitudinal studies should be designed to evaluate the long-term effects of gamification. As emphasized by Sailer and Homner [87], examining long-term effects is critically important for evaluating the sustainability of gamification approaches.
  - Reflection on Professional Practice: Research examining the reflection of concept image development on pre-service teachers’ professional practices should be designed. The dimension of transferring theoretical knowledge to practice emphasized by UNESCO is important in this context [24].
  - Mixed Method Research Designs: In future research, it is recommended to collect qualitative data from both experimental and control groups and conduct comparative analysis. In this research, qualitative data were collected only from experimental groups, creating a methodological limitation. Additionally, future studies should employ mixed-methods triangulation that combines concept image assessments with complementary measures across cognitive, affective, and behavioral dimensions. This multidimensional approach would provide more robust evidence of learning outcomes and help distinguish genuine conceptual restructuring from measurement artifacts or novelty effects. Future replications of Solomon four-group designs in educational research should aim to counterbalance program or departmental membership across pretest conditions, ensuring that pretest effects can be more cleanly separated from potential program-level influences. In addition, future studies should implement explicit cross-group contamination prevention protocols when applying experimental designs within shared institutional contexts. Specifically, researchers should formally instruct participants to refrain from sharing instructional materials or activity content across conditions, and should consider scheduling experimental and control group sessions in different academic terms where feasible to ensure more complete separation of conditions [42,44,45]. Additionally, rubric-based scoring procedures should incorporate formal blinding protocols in which response protocols are presented to all raters in anonymized and randomized order, with group membership and measurement time concealed, to minimize the risk of expectancy bias in scoring.
  - Multidimensional Measurement Approaches: Future research examining gamification effects on concept image development should incorporate complementary assessment methods beyond concept image questionnaires and reflective journals. Specifically, researchers should consider: (a) Performance-based assessments requiring application of curriculum knowledge to authentic teaching contexts (e.g., designing complete curriculum units, diagnosing curriculum design problems, adapting curricula for diverse learners) to evaluate knowledge transfer and deep understanding (b) Validated standardized instruments for affective dimensions, such as Intrinsic Motivation Inventory, Flow Experience Scale, or Teacher Self-Efficacy Scales, administered at pre-post time points to quantify motivational and confidence changes (c) Learning analytics and behavioral indicators from digital gamification platforms (time-on-task, attempt patterns, revision behaviors, help-seeking activities) to objectively verify self-reported engagement (d) Delayed post-tests (e.g., 3–6 months after intervention) to assess long-term retention and transfer of concept images to professional practice contexts. Such multidimensional assessment would enable triangulation of evidence across multiple data sources, strengthening confidence that observed improvements reflect sustainable learning rather than transient measurement artifacts.
- *Applications in Differe nt Contexts and Cultures:*
  - Application in Different Contexts: Similar studies should be conducted with different program types and groups of pre-service teachers. In particular, multinational research comparing the effects of gamification in different countries and cultural contexts will contribute to the evaluation of cross-cultural validity.
  - International Comparative Studies: International collaboration research comparing the effects of gamification approaches in teacher education systems of different countries should be designed. Such research will provide a better understanding of the cross-cultural validity and applicability of gamification. Teacher education standards of international organizations such as UNESCO [24] and OECD [20] can provide a common framework for these comparisons.
  - Cultural Context and Gamification Interaction: Research examining the role of different cultural values and educational traditions on gamification perceptions and effects should be designed. Hew et al.’s findings on cultural factors indicate the need for more detailed research in this area [33].
  - Research in the Context of Sustainable Development Goals: Longitudinal studies should be designed to examine the long-term effects of the gamification approach on pre-service teachers’ sustainable learning practices. Comparative research examining the relationship between gamification and sustainable development goals in different cultural contexts is gaining importance.
- *Examination of Gamification Strategies and Effects:*
  - Comparison of Gamification Elements: Studies comparing the effectiveness of different gamification elements should be conducted. Hamari et al.’s research on the effectiveness of gamification elements can guide these comparisons [28].
  - Curriculum Elements-Gamification Relationship: Research should be conducted to determine the most effective gamification strategies for each curriculum element. These studies can reveal how program understandings in different education systems shape gamification effects by including cross-cultural comparisons.
  - Examination of Possible Negative Effects: Research to identify and prevent possible negative effects of gamification should be conducted. The critical perspective initiated by Toda et al. [92] indicates the need for more research in this area.
  - Individual Characteristics and Gamification Interaction: Research examining the role of students’ individual characteristics such as learning styles, digital literacy levels, and gaming habits on gamification effectiveness should be designed. In this research, these variables were not controlled, but as Buckley et al. stated, individual differences can significantly shape the effectiveness of gamification [36]. Determining which gamification elements are more beneficial for students with different learning styles will contribute to the development of more inclusive gamification designs.
  - Sustainable Teacher Education Models: Mixed-method research should be designed to develop pre-service teachers’ skills in integrating curriculum literacy with sustainable education approaches. Assessment tools that evaluate the effectiveness of gamification elements in sustainable teacher education models should be developed.
- *Technology Integration:*
  - Technology-Supported Gamification: Research examining the effects of using different technology tools (virtual reality, augmented reality, mobile applications) in gamification-supported teaching should be designed. The integration of innovative technologies emphasized in the European Commission’s digital education action plan is important in this context [34].
  - Education Technologies and Gamification Integration: Research examining the integration of current education technologies with gamification approaches should be designed. The technology-supported learning goals stated in the European Commission’s “Digital Education Action Plan” framework can determine the orientation of such research [34].
- *Expansion of the Theoretical Framework:*
  - Expansion of the Theoretical Framework: More research combining concept image theory and the gamification approach should be conducted. Examining this integration in different disciplines and cultural contexts will contribute to the expansion of the theoretical framework.

This research has highlighted the importance of innovative approaches in teacher education by revealing the impact of gamification-supported teaching on pre-service teachers’ concept images. Gamification has been found to have meaningful potential, particularly in concretizing abstract concepts and creating active learning experiences. The potential for observing similar effects in different educational systems indicates that this approach can contribute to global teacher education standards. In the context of 21st-century teacher competencies emphasized by international organizations such as UNESCO and OECD, and in line with sustainable development goals, gamification-supported teaching approaches will contribute to the global dissemination of quality education.
